# Unraveling the genetic potential of native rice (*Oryza sativa* L.) landraces for tolerance to early-stage submergence

**DOI:** 10.3389/fpls.2023.1083177

**Published:** 2023-05-18

**Authors:** Aravindan Shanmugam, Kalaiarasan Manivelan, Konne Deepika, Gopal Nithishkumar, Viswanadhapalli Blessy, Raju Baskaran Monihasri, Dhanasekar Nivetha, Arunkamaraj Roshini, Palanivelu Sathya, Raman Pushpa, Rangarajan Manimaran, Kasirajan Subrahmaniyan, Datchinamoorthy Sassikumar, Ramalingam Suresh

**Affiliations:** ^1^ Centre for Plant Breeding and Genetics, Tamil Nadu Agricultural University (TNAU), Coimbatore, Tamil Nadu, India; ^2^ Department of Genetics and Plant Breeding, Agricultural College and Research Institute, Tamil Nadu Agricultural University (TNAU), Madurai, Tamil Nadu, India; ^3^ Department of Genetics and Plant Breeding, Anbil Dharmalingam Agricultural College and Research Institute, Tamil Nadu Agricultural University (TNAU), Trichy, Tamil Nadu, India; ^4^ Centre for Plant Molecular Biology and Biotechnology, Tamil Nadu Agricultural University (TNAU), Coimbatore, Tamil Nadu, India; ^5^ Tamil Nadu Rice Research Institute, Tamil Nadu Agricultural University (TNAU), Aduthurai, Tamil Nadu, India; ^6^ Sugarcane Research Station, Tamil Nadu Agricultural University (TNAU), Cuddalore, Tamil Nadu, India

**Keywords:** anaerobic germination tolerance, genetic diversity, grain type, rapid shoot elongation, response index

## Abstract

Direct-seeded rice (DSR) is a promising alternative to the traditional puddled rice system. It has become more popular among rice growers as a result of socioeconomic shifts and global climate change. Although DSR offers advantages, rice plants experience greater anaerobic stress at sowing from unpredicted rainfall. Rice is unique among cereals in its ability to germinate under anaerobiosis. The coleoptile of rice rapidly elongates above the water surface to obtain more oxygen and enhance vigorous seedling growth. A panel of 115 landraces and four check varieties were subjected to anaerobic stress with a water level of 10 cm for up to 15 days. The present study observed significant variation in anaerobic germination percentage (AGP) (10%–100%) and anaerobic vigor index (AVI) (150–4,433). Landraces *Karuthakar, Poovan samba, Mattaikar, Edakkal, Manvilayan*, and *Varappu kudainchan* were identified as genotypes tolerant to early water submergence. The shoot and root length of susceptible landraces were significantly lower than the tolerant landraces under hypoxia condition, implying that landraces with longer shoots and roots had a higher survival rate. The response index substantiated this. The results clearly show that tolerant and moderately tolerant landraces possessed higher mean values for root and shoot lengths than susceptible landraces. The landraces grouped under the long–bold category had superior AGP and AVI scores to other grain type groups. This raises the possibility that differences in kernel breadth, which is linked to grain type, could affect anaerobic germination potential. Molecular confirmation using gene-specific markers, *viz.*, DFR, TTP_G4, RM478, RM208, and RM24161, for which the polymorphic information content (PIC) value ranged from 0.36 (RM478) to 0.68 (RM206) suggests that this diverse panel of landraces must be assessed further using advanced molecular tools to precisely clarify the genetic mechanism behind this phenomenon. The tolerant landraces thus identified may become donors in breeding programs. The introduction of these traits would contribute to the development of rice varieties tolerant to anaerobic stress, resulting in sustainable yields. This solution could promote the DSR system across the world.

## Introduction

Direct-seeded rice (DSR) is a cultivation method that has gained popularity across rice-growing regions in recent years. Contributing factors to this trend the include reduced costs of production, reduced water demand, reduced labor requirements, and early maturity. Particularly in Asia, vast areas once covered by puddled transplant systems have adopted DSR systems (Ghosal et al., 2020). However, sudden rain and consequent flooding immediately after sowing or irrigation with saline water often produces anaerobic stress in plants cultivated by the DSR system (Islam et al., 2022). The seeds undergo hypoxia/anoxia, which in turn decelerates germination and seedling establishment due to the failure of adequate shoot and root formation. This phenomenon frequently results in complete crop failure (Ismail et al., 2009; Miro et al., 2017; Lal et al., 2018). Anaerobic stress has been identified as a major barrier to the broader adoption of DSR. Anaerobic stress results in poor germination and inadequate crop establishment, and is caused by unlevelled fields, unpredicted heavy rains after sowing, and severe weed infestation. Therefore, the wider adoption of DSR requires the development of rice varieties possessing the ability to germinate well under anaerobic stress and tolerate flooding at the time of germination.

Rice (*Oryza sativa* L.) is a semiaquatic plant (in contrast to other cereals), with its potential to germinate under hypoxic conditions due to its α-amylase genes (Guglielminetti et al., 1995; Hwang et al., 1999). These genes enable the seedlings to cope with the low levels of oxygen and sugar starvation that occur during hypoxia. Complete flooding at the time of germination leads to anoxia and results in poor or no germination. Rapid shoot elongation is a mechanism to escape anoxia. It contributes to anaerobic germination tolerance (AGT). In this way, seedlings can reach the water surface and diffuse O2 to elongate the roots and shoots (Ismail et al., 2009; Kretzschmar et al., 2015). Adaptive mechanisms and some of the key traits associated with anaerobic stress tolerance include anaerobic germination percentage (AGP) (Septiningsih et al., 2013; Barik et al., 2019), anaerobic vigor index (AVI) (Barik et al., 2019), response index (RI) (Islam et al., 2022), rapid elongation of the coleoptile (Kretzschmar et al., 2015), and carbohydrate reserves (Ella et al., 2011; Ismail et al., 2012).

Quantitative trait loci (QTL) mapping studies have identified numerous QTLs that govern major and minor effects of AGT (Jiang et al., 2006; Angaji et al., 2010; Septiningsih et al., 2013; Ghosal et al., 2019; Ghosal et al., 2020). QTL *qAG9-2* has been identified with 33.5% phenotypic variance on chromosome 9 (Angaji et al., 2010) and was fine mapped to the gene *OsTPP7* (Kretzschmar et al., 2015). This QTL is responsible for starch mobilization and favors the elongation of the coleoptile under anaerobic stress. Other QTLs were also identified as controlling AGT, such as *qAG7.1* with 31.7% phenotypic variance (Septiningsih et al., 2013) and *qAG7* with 22.3% phenotypic variance (Baltazar et al., 2014). To transfer the target loci into superior breeding lines, DNA markers closely linked with the target gene or QTL should be effective. Previously, DNA markers for AGT, such as RM 24161 and TTP_G4 for *AG1*, RM 3475 for *qAG1–2* (Angaji et al., 2010), DFR for *qAG9–2* (Kretzschmar et al., 2015), RM 478 for AG2 (Kim et al., 2019), and RM 341 and RM 206 for AGT (Reddy et al., 2015), were developed and are being used in marker-assisted breeding programs (Kim et al., 2019).

Genetic variation is the basis for any breeding program. It provides the breeder with pre-breeding material from which to choose (Palaniyappan et al., 2020). The landraces are called the ‘treasure of breeders’ as they harbor numerous varieties with diverse traits (i.e., in the case of this work, biotic and abiotic stress tolerance, nutrition, cooking, and quality traits). The genetic diversity of the rice germplasm, including the landraces, is relatively greater than other crop varieties. Being a primary center of origin for rice, India possesses more than 200,000 diverse rice varieties (Sathya, 2013). More than 10,000 popular indigenous rice accessions have been widely cultivated in India for millennia. *Munagada* (submerged) is a special landrace widely cultivated in the northern districts of Tamil Nadu, India. It can germinate in flood conditions and grow up to 3 feet in height (Stuart, 1895). The widespread cultivation of high-yield cultivars in a monoculture system has reduced the availability of most of the diverse indigenous rice landraces. Recent studies on anaerobic germination (AG) have identified several indigenous landraces with AGT such as MTU 1140 (Reddy et al., 2015); *Bausaganthi*, *Patadhan*, and *Basantichudi* (Barik et al., 2019); *Barkhe Tauli*, 498-2 A BR 8, *Jagli Boro*, *ParaNellu*, and Improved Blue Rose (Rauf et al., 2019); and *Vellai kavuni, Varappu kudaichan, Norungan*, and *Karuppu kavuni* (Mohanapriya et al., 2022). The application of intensive phenotyping protocols can validate the anaerobic germination tolerance of these genotypes. Breeders are encouraged to mine the existing diversity of rice varieties and identify novel potential donors for AGT.

The current study comprises the screening of a panel of 115 landraces along with four control varieties subjected to hypoxia to identify novel genotypes with AGT for further use in breeding programs. AGT-linked molecular markers have also been used in this study to screen the genotypes for allelic diversity associated with the targeted trait. We sought to identify traits associated with AG potential in the rice varieties, elucidate their physiological mechanisms, and document genetic and molecular variability so as to identify novel alleles.

## Materials and methods

A panel of 119 genotypes (**Supplementary Table 1**) containing 115 native landraces from south India and four control varieties, *viz.*, ‘FR13 A’, ‘IR 42’, ‘CO 43’, and ‘CO 43 Sub1’, was assembled for this study. Seeds of the selected indigenous landraces were collected from farmers, and raised and purified during the *Rabi* season in 2020 at the Tamil Nadu Rice Research Institute (TRRI), Aduthurai, Tamil Nadu, India (10°99'85"N and 79°48'01"E). These uniform healthy seeds were screened for AGT. Morphological traits such as kernel length (KL), kernel breadth (KB), length-to-breadth ratio (LBR), and 100-seed weight (HSW) were recorded for the selected genotypes. Grains of the selected genotypes were classified into different grain types as per the literature (Ramaiah, 1969) (**Supplementary Table 2**).

### Screening experiment for AGT

**Figure 1:**
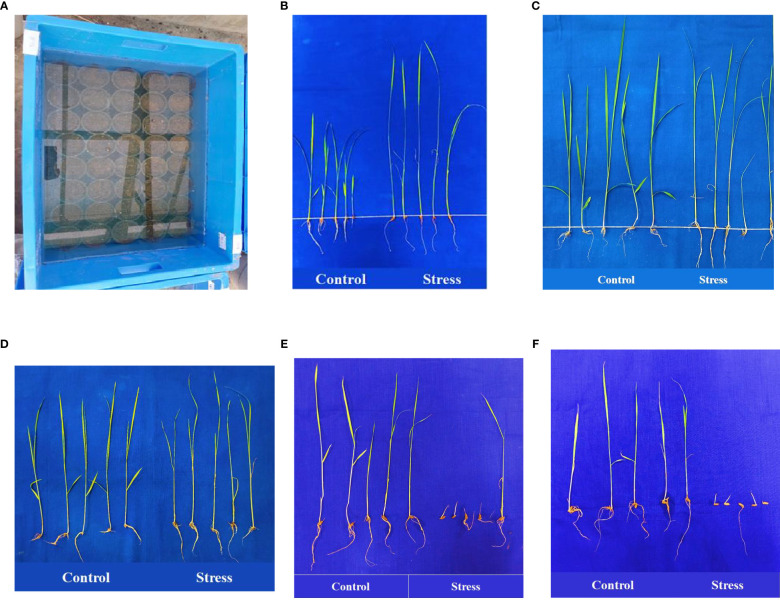
Anaerobic germination screening experiment **(A)**. Landraces *Poovan Samba* **(B)**, *Karuthakar* **(C)**, *Edakkal* **(D)**, ‘FR13 A’ **(E)**, and ‘IR 42’ **(F)** compared under controlled and stressed environments.


The anaerobic germination tolerance experiment was conducted on 20 September 2021 and 4 October, 2022 on the 119 selected genotypes at the Tamil Nadu Rice Research Institute during *Kharif*. Uniformly sized and well-filled seeds were surface sterilized with 0.1% HgCl_2_ solution for 5 min followed by 10 min of thorough washing with distilled water. The sterilized seeds were allowed to germinate under laboratory conditions in Petri dishes using wet germination paper. Five pre-germinated seeds were selected from each genotype, and were sown 1.0 cm below the surface of the soil in fine clay soil-filled cups (measuring 10 × 8 cm) in a plastic tray (sized 60 × 30 × 40 cm) with three replicates. Cups were then immediately submerged in water 10 cm above the soil surface, where they were held for 15 days (**Figure 1**). A control batch in which the soil surface was suitably moistened instead of completely submerged was established. After 15 days of hypoxia, AGP was measured as the number of emerged seedlings. For each replicate, the characteristics associated with seedling vigor were recorded for five control and five experimental seedlings, comprising shoot length (SL), root length (RL), shoot-to-root ratio (SRR), number of leaves (NOL), number of roots (NOR), fresh weight (FW), and dry weight (DW). Anaerobic Vigor Index (AVI) was calculated using the formula given by Barik et al. (2019):

(1) AVI=AGP×(shoot length + root length)

The anaerobic response index (RI) was calculated using the formula proposed by Islam et al. (2022).


(2) RI = shoot length (submerged) - shoot length (control)

### Molecular characterization

Total genomic DNA was isolated for all the genotypes using the CTAB (cetyltrimethylammonium bromide) method (Murray and Thompson, 1980). The quantity of the DNA was determined using a spectrophotometer based on an A260/A280 ratio. DNA was diluted with molecular-grade water before performing PCR and conducting electrophoretic analysis.

The allelic status of the AGT genotypes was examined using inserations and deletions (InDels) and simple sequence repeats (SSR) markers linked to AGT. The details of the markers used in this genotypic analysis are given in **Table 1**. PCR amplification was conducted in a Veriti master cycler with 10 µL of PCR reaction mixture containing 1 µL (25 ng/µL) of genomic DNA, 1 µL of SSR primer (2 mM), and 8 µL of the commercial PCR master mix (1×). Initial denaturation was at 94°C for 5 min, which was then followed by 35 cycles of denaturation at 94°C for 30 s, annealing at 55°C for 30 s, extension at 72°C for 1 min, and a final extension at 72°C for approximately 10 min. The amplified PCR products were then resolved in a 2.5% agarose gel and documented using the Syngene G: BOX F3 system. Amplified bands of each marker locus were scored according to the presence or absence of the bands in each of the studied rice genotypes. Genetic diversity parameters, such as the number of alleles and polymorphic information content (PIC), were calculated using the PowerMarker Ver3.25 program (Lu et al., 2005).

**Table 1:** Detailed list of gene-specific markers for anaerobic germination tolerance.

Marker	R gene	Type	Sequence (5′−3′)	Reference
**RM 24161**	*AG1*	SSR	F: GTATGGCGAGACCCTACAGACC R: GACCCACTTAATGTGTCACAAGG	Kim et al., 2019
**RM 478**	*AG2*	SSR	F: GGGTGGAGTGTAATAATAGCAAGC R: AACACGTCCAAAGTCACAGAGC	Kim et al., 2019
**TTP_G4**	*AG1*	InDel	F: AATGGTGTCCACATTGCAGA R: GCATTGATCTTCCTCTTGTGC	Kim et al., 2019
**DFR**	*AG1*	InDel	F: CCACCATGATGTAGTTCAGTTGTGAAC R: CACCGTTAAAATCGGCCGTTAG LB: CGGCTTCGTCTTCACCTGAAC	Kretzschmar et al., 2015
**RM 206**	*qAG11*	SSR	F: ATCGATCCGTATGGGTTCTAGC R: GTCCATGTAGCCAATCTTATGTGG	Reddy et al., 2015

### Statistical analysis

An analysis of variance (ANOVA) for traits associated with AG potential was conducted with R studio software (Racine, 2012) using the “agricolae” package (de Mendiburu and de Mendiburu, 2019). The Newman–Keuls test (Newman, 1939; Keuls, 1952) was used to compare the group means of the grain type and AGT, using the ‘agricolae’ package. Correlation between traits was estimated using the ‘corrplot’ package (Wei et al., 2017). Principal component analysis (PCA) was conducted using the ‘FactoMineR’ and ‘factoectra’ packages. Phenotypic clustering was performed using the Ward.D2 method with the help of Gower’s distance matrix (Murtagh and Legendre, 2014), whereas the significance of the cluster means was determined using the Newman–Keuls test. The Nei distance matrix was estimated and the Ward.D2 method of clustering was used to group the genotypes based on their molecular information. Based on *per se* performance, promising genotypes for AGT were identified.

## Results

### Estimates of the variance components, genetic variability, and heritability

This study investigated variations in the physical characteristics and anaerobic germination potential of native south Indian landraces under normal and hypoxic conditions to assess the tolerance of these landraces to flooding at the time of germination. The variance analyses conducted on the anaerobic germination-associated traits of the 119 genotypes revealed highly significant genotypic variance within each individual year and in the pooled data over the years, except in the case of the fresh and dry weights of the seedlings (**Table 2**). A significant variation (*p*< 0.01) was found for AGP, ranging from 10% (*Samba mosanam*) to 100% (*Karuthakar*), and AVI ranged from 150 (*Samba massanam*) to 4,433 (*Poovan samba*) (**Table 3**). Shoot lengths of the germinating seedlings varied from 5.14 cm (*Samba mosanam*) to 35.76 cm (*Poovan samba*), whereas the root length varied from 1.69 cm (*Salem samba*) to 11.63 cm (*Iravai pandi*). The response index ranged from –1.92 (*Thillainayagam*) to 17.42 (*Edakkal*). The traits SRR (1.09–12.29), NOL (0.67–2.56) and NOR (2.40–11.41) showed considerable variation. Significant variations were found in morphological characteristics (KL, KB, and LBR) and test seed weight (*p*< 0.01) among the landraces studied. The KL, KB, LBR, and grain weight ranged from 4.00 (*Kothamalli samba*) to 9.70 mm (*Chinnar*), 1.60 (*Jai Sri Ram*) to 3.80 mm (*Thondi*), 1.67 (*Kothamalli samba*) to 3.95 (*Chinna punchai*), and 0.78 (*Thulasi vasanai*) to 3.65 g (*Thondi*), respectively, among the studied landraces.

**Table 2:** Estimated variance components for traits with anaerobic germination percentage of indigenous rice landraces evaluated in 2021 and 2022 at Tamil Nadu Rice Research Institute, Aduthurai, Tamil Nadu, India.

Trait	Season I		Season II		Pooled		
	σ^2^g^#^	σ^2^error	σ^2^g	σ^2^error	σ^2^g	σ^2^error	σ^2^ge
**AGP (%)**	1,220.83^**^	389.33	2,034.51^**^	2.13	2,878.28^**^	195.73	377.06^**^
**AVI**	2,304,149.05^**^	573,976.78	4,781,244.40^**^	3,934.53	6,068,623.39^**^	288,955.65	1,016,770.06^**^
**SL (cm)**	98.81^**^	29.87	227.76^**^	0.26	225.03^**^	15.06	101.54^**^
**RL (cm)**	12.26^**^	4.16	30.82^**^	0.05	25.78^**^	2.10	17.30^**^
**RI**	120.29^**^	29.87	260.06^**^	0.26	278.74^**^	15.06	101.61^**^
**SRR**	20.87^**^	15.57	4.00^**^	0.01	13.00^**^	7.79	11.87^**^
**NOL**	0.42^**^	0.22	0.47^**^	0.00	0.48^**^	0.11	0.41^**^
**NOR**	11.98^**^	3.06	10.54^**^	0.02	16.61^**^	1.54	5.92^**^
**FW (g)**	0.020	0.020	0.023^**^	0.00001	0.024^**^	0.010	0.019^**^
**DW (g)**	0.005	0.006	0.013^**^	0.0001	0.011^**^	0.003	0.007^**^

**significant at *p*-value< 0.01; ^#^ σ^2^g, genotypic variance; σ^2^error, error variance; σ^2^ge, genotypic × environment variance. AGP, anaerobic germination percentage; AVI, anaerobic vigor index; SL, shoot length; RL, root length; RI, response index; SRR, shoot-to-root ratio; NOL, number of leaves; NOR, number of roots; FW, fresh weight; DW, dry weight.

**Table 3:** Genetic variability parameters of studied traits of indigenous rice landraces germinating under anaerobic conditions.

Trait	Range	Mean	GCV (%)	PCV (%)	${\text{h}}_{\text{bs}}^{2}$	GA	GAM	SED
**AGP (%)**	10 to 100	59.80 ± 2.01	50.00	55.20	82.04	50.54	84.51	8.08
**AVI**	150 to 4433	1953.26 ± 92.19	71.06	76.20	86.96	2,486.37	127.29	310.35
**SL (cm)**	5.14 to 35.76	22.78 ± 0.56	36.73	40.49	82.29	14.18	62.26	2.24
**RL (cm)**	1.69 to 11.63	6.86 ± 0.19	40.95	46.09	78.95	4.57	66.60	0.84
**RI**	–11.92 to 17.42	3.05 ± 0.62	30.75	33.28	85.37	16.49	540.89	2.24
**SRR**	1.09 to 12.29	3.85 ± 0.13	34.26	80.23	18.23	0.49	12.87	1.61
**NOL**	0.67 to 2.56	1.88 ± 0.03	18.58	25.64	52.49	0.38	20.08	0.19
**NOR**	2.40 to 11.41	6.20 ± 0.15	36.12	41.29	76.52	3.53	56.93	0.72
**FW (g)**	0.03 to 0.43	0.15 ± 0.01	46.55	83.50	31.08	0.04	29.80	0.06
**DW (g)**	0.01 to 0.31	0.09 ± 0.00	60.86	88.15	47.67	0.05	59.77	0.03
**KL (mm)**	4 to 9.70	6.61 ± 0.10	16.19	16.43	97.19	2.14	32.42	0.15
**KB (mm)**	1.60 to 3.80	2.41 ± 0.04	18.20	18.39	97.86	0.88	36.68	0.05
**LBR**	1.67 to 3.95	2.79 ± 0.04	15.14	15.38	96.95	0.84	30.24	0.06
**HSW (g)**	0.78 to 3.65	2.36 ± 0.06	27.53	27.69	98.85	1.32	56.05	0.06

GCV, genotypic coefficient of variation; PCV, phenotypic coefficient of variation; ${\text{h}}_{\text{bs}}^{2}$ , broad-sense heritability; GA, genetic advance; GAM, genetic advance as a percentage of mean; SED, standard error deviation. AGP, anaerobic germination percentage; SL, shoot length; RL, root length; RI, response index; SRR, shoot-to-root ratio; NOL, number of leaves; NOR, number of roots; FW, fresh weight; DW, dry weight; AVI, anaerobic vigor index; KL, kernel length; KB, kernel breadth; LBR, length-to-breadth ratio; HSW, hundred-seed weight.

The phenotypic coefficient of variation (PCV), genotypic coefficient of variation (GCV), heritability, and genetic advance (GA) were evaluated for the 119 rice genotypes. The PCV values ranged from 15.38% (LBR) to 88.15% (DW), whereas GCV ranged from 15.14% (LBR) to 71.06% (AVI). The PCV value was higher than the GCV value for all studied traits. This finding demonstrates the predominance of environmental interaction in determining the expression of these traits. AGT-associated traits, such as AGP, AVI, SL, and RL, had a high PCV coupled with strong GCV values. Broad-sense heritability varied widely from 18.23% to 98.85%. Traits associated with AG potential, such as AGP, AVI, RI, SL, and RL, along with seed morphological traits such as KL, KB, LBR, and HSW, had high heritability. Genetic advance as a percentage of means ranged from 12.87% (SRR) to 540.89% (RI). Traits such as AGP, AVI, SL, RL, RI, and grain weight displayed a high genetic advance as a percentage of mean (GAM) and high heritability.

### Grain morphology and seedling growth characteristics

Morphological characteristics of the grain, *viz*., KL, KB, LBR, and HSW, varied widely (**Table 4**). The studied landraces were grouped into five groups, *viz*., short–bold, short–slender, medium–slender, long–bold and long–slender (Ramaiah, 1969). Out of the total 119 genotypes, 10 were grouped under the short–bold type, whereas nine landraces were classified under the short–slender grain type. Thirteen landraces were grouped under the medium–slender type and 24 landraces were placed in the long–slender group. The majority, i.e., 63 landraces, were classified under the long–bold grain type.

**Table 4:** Pooled mean values of traits associated with anaerobic germination potential and grain morphology of native rice landraces.

Genotypes	AGP	AVI	SL	RL	RI	SRR	NOL	NOR	FW	DW	KL	KB	LBR	HSW	GT
***Karuthakar***	100.00	4,206	33.53	8.53	14.53	5.32	2.5	5.3	0.08	0.03	6.4	2.2	2.91	2.63	Long–bold
***Edakkal***	96.67	4,046	31.80	9.75	17.42	3.47	2.1	10.4	0.09	0.03	5	2	2.50	2.63	Long–bold
***Mattaikar***	96.67	4,117	32.36	9.70	4.35	3.42	2.0	7.2	0.17	0.10	7.2	3	2.40	2.31	Long–bold
***Poovan samba***	96.67	4,433	35.76	10.14	14.98	3.68	2.2	4.8	0.19	0.12	6	3	2.00	3.09	Long–bold
***Mandamaranellu***	93.33	3,641	30.43	8.57	7.23	4.15	2.1	8.0	0.16	0.10	6	2.5	2.40	2.44	Long–bold
***Manvilayan***	93.33	3,388	26.85	9.33	6.85	2.90	1.9	7.0	0.17	0.12	6.3	2.6	2.42	3.02	Long–bold
***Varappu kudainchan***	93.33	3,561	30.77	7.38	0.71	4.61	2.0	6.6	0.11	0.07	5.4	2.3	2.35	2.39	Short–bold
***Kaan***	90.00	3,422	28.49	9.48	1.45	3.33	2.3	10.1	0.17	0.09	6.5	2.5	2.60	2.52	Long–bold
***Katta samba***	90.00	3,207	28.15	7.66	7.58	3.89	2.1	6.9	0.12	0.06	8	3.1	2.58	2.44	Long–bold
***Mohini Samba***	90.00	3,040	25.11	8.69	13.81	3.08	1.9	6.6	0.13	0.07	6.4	2.7	2.37	2.41	Long–bold
***Varisurian***	90.00	3,340	28.71	8.50	4.13	3.83	1.6	4.4	0.10	0.04	7.7	3.1	2.48	2.49	Long–bold
***Iravai Pandi***	86.67	3,322	26.38	11.63	12.60	2.29	2.3	5.4	0.22	0.17	6	2.5	2.40	2.49	Long–bold
***Karuppu Nel***	86.67	3,229	31.16	5.64	12.67	6.20	1.9	5.4	0.10	0.06	8.5	3.2	2.66	2.97	Long–bold
***Kattai kar***	86.67	2,987	28.20	6.20	9.08	5.69	2.3	5.3	0.09	0.06	6.2	2	3.10	2.45	Long–slender
***Ottadai***	86.67	3,466	29.49	10.56	7.09	2.91	1.8	5.3	0.11	0.06	6	2.4	2.50	2.95	Long–bold
***Navara***	83.34	3,218	31.13	7.78	3.43	5.55	2.0	6.2	0.13	0.06	6.1	2.3	2.65	2.25	Long–bold
***Gedumani***	83.33	3,208	30.56	7.89	8.90	5.04	2.2	4.4	0.09	0.05	8.2	2.1	3.90	2.89	Long–slender
***Kottanel***	83.33	3,185	30.85	7.62	13.68	5.19	2.1	6.1	0.12	0.07	8	3	2.67	2.45	Long–bold
***Poongar***	83.33	3,057	29.65	7.87	8.00	4.26	1.9	4.4	0.13	0.09	7	2.1	3.33	3.05	Long–slender
***Uppu Milagai***	83.33	3,130	28.68	8.91	6.76	5.30	2.0	5.1	0.11	0.06	5.5	2.1	2.62	2.33	Medium–slender
***Vasaramundan***	83.33	3,166	29.81	8.68	2.03	3.76	1.8	7.2	0.17	0.08	6.7	2.5	2.68	3.42	Long–bold
***Norungan***	83.33	2,340	23.14	5.46	1.65	4.22	1.9	5.6	0.13	0.07	6.7	2.5	2.68	3.44	Long–bold
***Thengai poo samba***	80.00	2,658	27.60	5.45	4.21	5.23	1.9	5.3	0.08	0.03	6.3	2.1	3.00	2.32	Long–slender
***Koom vazhai***	80.00	2,834	28.16	7.12	1.35	6.05	1.9	5.4	0.12	0.05	9.6	3.3	2.91	3.21	Long–bold
***Chitti mutyalu***	80.00	2,279	20.68	7.94	1.75	2.94	1.8	7.6	0.16	0.10	5.7	1.9	3.00	0.96	Short–bold
***Gandakasala***	80.00	2,910	27.45	8.80	7.30	3.17	2.0	6.6	0.13	0.06	8	2.8	2.86	2.35	Long–bold
***Kuliadichan***	80.00	2,706	25.07	7.17	6.32	4.34	2.5	5.4	0.08	0.03	6.5	2.4	2.71	2.99	Long–bold
***Thandi palliyan***	80.00	3,005	29.40	8.17	9.48	4.98	1.9	7.5	0.20	0.09	6.6	2.5	2.64	3.17	Long–bold
***Chinna Punchai***	76.67	2,930	29.27	8.50	2.73	3.61	2.6	7.8	0.13	0.07	7.9	2	3.95	2.92	Long–slender
***Kallundai***	76.67	2,769	28.05	8.51	9.11	3.57	2.3	5.6	0.13	0.08	6	2.5	2.40	3.01	Long–bold
***Kandhasali***	76.67	2,528	24.40	8.22	9.26	3.38	1.9	3.5	0.08	0.04	6.2	2.2	2.82	2.21	Long–bold
***Nootripathu***	76.67	2,208	20.50	8.83	–1.25	2.12	1.5	7.1	0.13	0.07	8	3.2	2.50	2.59	Long–bold
***Sowattara samba***	76.67	3,161	33.38	8.60	13.20	4.91	1.8	5.0	0.10	0.04	6.7	2.6	2.58	1.63	Long–bold
***Chenellu***	76.67	2,585	25.37	8.39	5.65	3.18	1.5	9.7	0.12	0.07	6.4	2.2	2.91	2.76	Long–bold
***Chinkini kar***	76.67	3,277	31.77	11.01	14.78	3.07	1.9	10.3	0.18	0.11	6.4	2.5	2.56	3.37	Long–bold
***Kaliyan Samba***	76.67	2,757	26.31	9.92	5.63	2.74	1.9	9.0	0.21	0.15	6.1	2.4	2.54	2.77	Long–bold
***Karnel***	76.67	2,200	18.82	9.49	–0.57	2.06	1.6	6.0	0.15	0.08	8	2.1	3.81	1.60	Long–slender
***Kudavaraghai***	76.67	3,000	30.91	7.99	10.25	4.34	2.0	5.2	0.16	0.12	8	3.4	2.35	3.10	Long–bold
***Kuruvai kalanjiyam***	76.67	3,025	32.24	7.33	12.25	4.70	2.2	6.4	0.23	0.12	8.6	3.1	2.77	2.79	Long–bold
***Vellai kavuni***	76.67	2,475	22.64	9.00	–8.03	2.83	2.0	5.8	0.17	0.10	8.5	2.4	3.54	2.22	Long–slender
***Valan***	73.34	2,639	27.30	9.35	13.10	2.96	2.0	6.7	0.08	0.04	6.2	2.4	2.58	3.00	Long–bold
***Kuthala samba***	73.33	2,187	20.97	7.89	8.57	2.81	1.6	6.4	0.08	0.06	5.7	2.5	2.28	1.89	Short–bold
***Athur kichadi***	73.33	2,512	23.82	10.51	6.85	2.38	2.0	6.6	0.18	0.13	6.7	2.9	2.31	1.71	Long–bold
***Koduvaliyan***	73.33	2,387	25.61	6.77	10.10	4.72	1.8	3.4	0.10	0.06	7.7	3.3	2.33	2.12	Long–bold
***Kullakkar***	73.33	2,631	28.69	7.09	7.82	4.24	2.0	6.5	0.22	0.12	6.6	2.7	2.44	2.32	Long–bold
***Mysore malli***	73.33	2,449	24.71	9.05	4.80	2.78	2.1	6.6	0.08	0.03	6.3	2.1	3.00	2.02	Long–slender
***Soora kuruvai***	73.33	2,963	31.85	7.92	3.46	4.69	1.9	5.5	0.16	0.09	6.2	2.5	2.48	3.02	Long–bold
***Mallikar***	70.00	2,295	25.50	8.63	–1.65	3.01	1.9	7.6	0.24	0.14	6.5	2.5	2.60	2.93	Long–bold
***Sengalpattu sirumani***	70.00	2,101	22.03	8.31	11.20	2.65	1.7	5.0	0.21	0.13	7.2	3.1	2.32	1.76	Long–bold
***Pal Kichadi***	66.67	1,998	22.03	8.28	5.96	2.72	2.0	7.5	0.10	0.07	6.2	2.3	2.70	3.36	Long–bold
***Karuppu kavuni***	66.67	2,219	25.48	6.70	9.90	8.73	2.0	6.8	0.17	0.11	6.8	2.5	2.72	2.68	Long–bold
***Kalanamak***	63.33	1,948	23.79	7.06	6.23	3.67	2.0	7.0	0.13	0.08	6.2	1.8	3.44	2.59	Long–slender
***Mullampunchan***	63.33	1,703	20.55	5.21	1.00	4.19	2.0	5.9	0.17	0.08	5.9	2.6	2.27	2.88	Short–bold
***Rajamannar***	63.33	1,503	16.62	6.87	–2.13	2.58	2.0	7.1	0.07	0.01	5.7	2	2.85	1.82	Medium–slender
***Thondi***	63.33	1,793	19.87	7.49	4.26	2.64	1.9	8.4	0.12	0.08	9	3.8	2.37	3.65	Long–bold
***Madu muzhunki***	60.00	2,318	30.68	7.45	6.40	4.31	2.2	5.5	0.19	0.12	6.7	2.4	2.79	2.80	Long–bold
***Aanai komban***	60.00	2,159	27.85	8.06	3.99	3.46	2.0	7.8	0.17	0.09	8.3	2.4	3.46	3.13	Long–slender
***Chandai kar***	60.00	1,498	19.43	5.83	6.21	3.42	2.0	6.1	0.14	0.08	8.3	3	2.77	2.23	Long–bold
***Rasakatam***	60.00	1,549	20.05	5.47	0.04	3.97	1.5	5.2	0.12	0.07	5.8	2	2.90	1.80	Medium–slender
***Salem sannam***	60.00	1,625	20.05	6.83	2.03	4.17	1.8	4.4	0.06	0.04	7.7	2.7	2.85	2.36	Long–bold
***Sivappu malli***	60.00	1,760	21.24	7.39	–0.58	2.83	2.0	7.3	0.11	0.07	5.6	2.2	2.55	1.91	Medium–slender
***Sugandni samba***	56.67	1,614	21.84	5.48	9.01	4.87	2.1	5.6	0.08	0.04	6.3	1.7	3.71	1.24	Long–slender
***Adukkan***	56.67	2,034	24.21	10.49	13.53	2.43	1.8	6.1	0.14	0.10	6.5	2.2	2.95	2.17	Long–bold
***Mappilai samba***	56.67	1,782	24.53	7.70	1.54	3.34	1.9	6.9	0.16	0.15	6.7	2.5	2.68	3.27	Long–bold
***Rathasali***	56.67	1,180	14.94	6.30	3.80	2.38	1.9	4.2	0.08	0.03	5.5	1.8	3.06	1.16	Short–bold
***Jaya***	56.67	1,931	24.95	8.93	6.40	2.79	1.7	8.2	0.18	0.12	6.4	2.4	2.67	2.83	Long–bold
***Kothamalli samba***	53.34	1,244	19.74	6.63	–4.47	2.97	1.8	7.8	0.12	0.07	4	2.4	1.67	1.75	Short–bold
***Sanka samba***	53.34	1,581	23.15	6.97	7.18	4.69	1.5	2.4	0.14	0.09	5.7	2.2	2.59	2.16	Medium–slender
***Illupai poo Samba***	53.33	1,535	24.90	3.66	8.87	7.44	1.9	3.1	0.43	0.11	6.1	1.9	3.21	1.52	Long–slender
***Milagu samba***	53.33	1,177	17.78	4.32	1.99	4.76	1.7	4.3	0.06	0.03	6	2.6	2.31	1.56	Long–bold
***Sembalai***	50.00	1,532	24.61	6.17	8.73	5.92	1.4	4.7	0.17	0.10	6	2.1	2.86	2.34	Long–bold
***Chithirai Kar***	50.00	1,068	15.61	5.18	–9.64	2.96	2.1	5.8	0.23	0.12	5.9	2.5	2.36	3.26	Short–bold
***Kaatu Ponni***	50.00	1,627	22.26	7.79	–4.94	2.85	1.7	8.8	0.14	0.16	6.6	2	3.30	2.28	Long–slender
***Kaivara samba***	50.00	1,442	20.07	7.32	0.40	2.72	2.0	7.5	0.15	0.08	5.9	1.9	3.11	3.15	Short–bold
***Karimbalan***	50.00	1,774	26.59	7.84	5.95	3.58	2.0	11.4	0.21	0.11	8.2	3	2.73	3.05	Long–bold
***Kichali samba***	50.00	1,440	19.20	7.69	–9.87	2.50	2.0	8.6	0.18	0.12	6.2	1.9	3.26	1.92	Long–slender
***Seeraga samba***	50.00	1,465	21.99	7.28	6.76	3.14	2.4	5.6	0.12	0.06	5	1.8	2.78	1.15	Medium–slender
***Thanga samba***	50.00	1,678	24.87	8.43	0.27	3.04	1.9	7.2	0.15	0.11	5.7	2.2	2.59	2.42	Medium–slender
***Chinna ponni***	50.00	1,422	20.84	4.89	–11.20	4.65	2.1	5.6	0.09	0.05	7.4	2.5	2.96	2.09	Long–bold
***Bhavani***	50.00	1,657	23.17	7.02	10.84	12.29	1.9	6.6	0.16	0.10	6.8	2	3.40	2.37	Long–slender
***Purple puttu***	50.00	1,564	21.96	8.20	8.21	2.67	2.2	5.3	0.13	0.10	8.7	2.3	3.78	1.77	Long–slender
**‘FR13 A’**	50.00	847	12.68	5.22	–9.33	2.41	2.1	7.4	0.11	0.06	7.8	3.2	2.44	3.03	Long bold
***Revathi***	46.67	1,361	23.64	6.94	–6.76	3.41	1.9	6.6	0.17	0.11	5.7	1.8	3.17	1.49	Short–bold
***Kattu vanibam***	46.67	1,597	26.94	7.06	8.92	4.07	1.8	7.8	0.24	0.13	5.7	2.6	2.19	3.61	Short bold
**‘CO 43 Sub1’**	46.67	804	12.14	3.86	–5.30	3.57	1.8	4.8	0.15	0.08	7.9	2.9	2.72	2.14	Long–bold
***Kottara samba***	46.67	1,229	16.20	4.57	–2.40	3.61	1.6	5.3	0.21	0.31	6	2.6	2.31	2.84	Long–bold
***Pommi***	46.67	1,446	20.69	8.28	3.06	2.50	1.9	9.3	0.12	0.08	5.7	2	2.85	1.49	Medium–slender
**‘CO 43’**	46.67	861	12.39	3.04	–5.68	3.77	2.0	4.6	0.14	0.08	7.8	2.7	2.89	2.10	Long–bold
***Savul samba***	43.33	1,059	18.59	5.48	–2.99	3.49	1.9	6.6	0.11	0.08	7.4	2.6	2.85	1.95	Long–bold
***Melaki***	43.33	995	18.34	2.93	–2.67	6.47	1.6	4.4	0.08	0.04	4.5	2.4	1.88	1.56	Short–bold
***Aarupatham kuruvai***	40.00	934	19.14	3.89	–7.91	5.97	1.7	4.7	0.11	0.07	7.2	2.9	2.48	2.18	Long–bold
***Thulasi vasanai***	40.00	1,057	16.57	4.61	1.24	3.88	1.9	7.2	0.19	0.09	4.1	1.7	2.41	0.78	Short–bold
***Vasanai seeraga samba***	40.00	1,050	17.29	4.27	6.13	5.13	1.6	4.4	0.11	0.07	5.6	2	2.80	0.94	Medium–slender
***Aathur kichadi samba***	36.67	842	14.71	5.98	–8.72	2.34	2.1	5.6	0.26	0.20	5.8	1.9	3.05	1.61	Short–bold
**‘GEB-24’**	36.67	1,254	25.96	5.64	2.43	5.39	1.8	7.1	0.22	0.17	5.9	2	2.95	2.46	Medium–slender
***Kaatu samba***	36.67	1,052	19.15	6.32	–11.13	3.10	2.0	6.9	0.22	0.14	6.4	1.9	3.37	1.42	Long–slender
***Karudan samba***	36.67	1,046	19.02	3.73	0.79	5.88	1.2	4.8	0.24	0.20	5.6	2.4	2.33	2.31	Short–bold
***Mutrina Samba***	36.67	921	14.84	4.07	–7.04	4.41	1.4	4.1	0.14	0.08	6.2	2	3.10	1.76	Long–slender
***Pisini***	36.67	762	14.63	2.54	–6.97	6.52	1.8	3.4	0.07	0.04	6.7	2.9	2.31	3.33	Long–bold
***Thillainayagam***	36.67	1,050	18.03	5.86	–11.92	3.27	2.1	7.0	0.08	0.05	7.5	2.3	3.26	1.88	Long–slender
***Thirupathisaram***	36.67	978	17.15	4.26	–4.19	3.95	2.3	6.4	0.40	0.10	5.9	2.3	2.57	2.46	Medium–slender
***Altera***	36.67	877	14.70	5.36	–0.71	2.71	1.9	6.1	0.25	0.17	6.5	2.4	2.71	2.64	Long–bold
***Athira***	36.67	1,093	18.93	7.45	3.27	2.35	1.7	8.2	0.22	0.11	5.6	1.8	3.11	3.08	Short–bold
***Ottadaiyan***	36.67	1,183	25.41	5.94	15.12	5.02	1.7	7.1	0.09	0.05	8	3.1	2.58	2.80	Long–bold
***Gopal bhog***	36.67	1,023	20.16	7.17	–0.17	2.94	1.5	7.4	0.13	0.08	6.5	2	3.25	1.54	Long–slender
***Ganga***	33.34	929	16.71	5.73	0.50	2.66	1.7	7.1	0.12	0.08	7.2	2.1	3.43	1.93	Long–slender
***Vellai chithirai kar***	33.33	864	17.16	6.09	–4.21	2.78	1.3	6.5	0.19	0.10	5.6	2.2	2.55	1.05	Medium–slender
***Vadan samba***	30.00	846	21.81	5.77	2.11	3.93	2.2	6.8	0.10	0.05	5.5	1.8	3.06	2.57	Short–bold
***Chinnar***	30.00	722	16.44	4.98	–7.48	3.22	1.4	8.1	0.22	0.13	9.7	3.4	2.85	2.21	Long–bold
***Jai Sri Ram***	30.00	667	14.83	4.49	6.85	3.28	1.3	5.6	0.20	0.14	5.6	1.6	3.50	1.28	Short–bold
***Manjal ponni***	30.00	662	15.38	3.62	–0.29	4.05	1.8	3.1	0.09	0.04	6.2	2.2	2.82	1.61	Long–bold
***Thuyamalli***	30.00	661	17.67	4.60	–3.28	4.31	1.7	5.4	0.10	0.05	6.4	2	3.20	1.70	Long–slender
***Saysree***	26.67	739	20.17	6.05	0.89	3.45	1.8	6.2	0.24	0.12	5.6	2.3	2.43	2.16	Short–bold
***Kichadi samba***	23.33	486	16.69	3.28	0.33	5.29	1.9	5.0	0.20	0.11	5.5	2	2.75	1.66	Medium–slender
***Sivappu chithiraikar***	16.67	608	14.16	3.47	–4.49	2.30	2.0	6.6	0.03	0.02	6.3	2.6	2.42	3.55	Long–bold
**‘IR42’**	13.34	279	12.30	3.18	–3.30	3.27	1.8	4.2	0.12	0.06	8.9	2.4	3.71	2.58	Long–slender
***Maranellu***	13.33	260	9.18	3.80	–3.70	1.64	1.5	3.8	0.06	0.03	7	2	3.50	2.28	Long–slender
***Salem samba***	13.33	250	7.31	1.69	–9.23	2.19	0.7	4.3	0.08	0.05	5.8	1.9	3.05	1.77	Short–bold
***Samba massanam***	10.00	150	5.14	2.35	–2.33	1.09	1.1	2.5	0.03	0.01	6.5	2.4	2.71	2.08	Long–bold

Landraces *Karuthakar, Poovan samba, Mattaikar, Edakkal, Manvilayan*, and *Varappu kudainchan* were identified as genotypes tolerant to early water submergence and may become donors in breeding programs. AGP, anaerobic germination percentage; AVI, anaerobic vigor index; SL, shoot length; RL, root length; RI, response index; SRR, shoot-to-root ratio; NOL, number of leaves; NOR, number of roots; FW, fresh weight; DW, dry weight; KL, kernel length; KB, kernel breadth; LBR, length-to-breadth ratio; HSW, 100-seed weight; GT, grain type.

Anaerobic germination percentage is a key trait to identify the genotypes that are tolerant to hypoxia under the DSR cultivation system. *Samba masanam* possessed the least AGP (10%), followed by *Salem samba*, *Maranellu*, and ‘IR 42’ (13.33%). *Karuthakar* displayed the maximum AGP (100%) after 15 days of hypoxia. On the basis of AGP, the landraces were divided into four categories, as proposed by Barik et al. (2019). Landraces displaying an AGP of ≥ 90% were categorized as “tolerant landraces”; 11 landraces were grouped under this category. With an AGP of 71%–89%, 36 landraces were grouped as “moderately tolerant landraces”. The 43 landraces with an AGP of 41%–70% were identified as “moderately susceptible landraces”. A total of 29 landraces had an AGP of ≤ 40% and were classified as “susceptible landraces”. Control varieties ‘FR13 A’, ‘CO 43’, and ‘CO 43 Sub1’ were found to possess an AGP of 41%–70% and were identified as moderately susceptible, whereas ‘IR 42’ was categorized as susceptible with an AGP of 13.34%. Under non-stressed, aerobic-controlled conditions, all 119 genotypes achieved 100% germination.

Shoot elongation under anaerobic stress is another important adaptive mechanism that helps emerging seedlings tolerate early-stage submergence. The response index (RI) is generally used to identify the genotypes with shoot elongation under stress compared with the control environment. RI values ranged from –11.92 (*Thillainayagam*) to 17.42 (*Edakkal*). Thirty-six landraces scored as susceptible or moderately susceptible had RI values less than zero. This finding implies a reduction of shoot length under hypoxia. In addition, AVI was calculated based on a seedling’s growth characteristics and AGP. Landraces *Poovan samba* (4,433), *Karuthakar* (4,206), *Mattaikar* (4,117), and *Edakkal* (4,046) had higher AVI values than the susceptible landraces *Samba massanam* (150), *Salem samba* (250)*, Maranellu* (260), and ‘IR 42’ (279).

### Mean comparison of the grain groups vs. tolerance reaction

**Figure 2:**
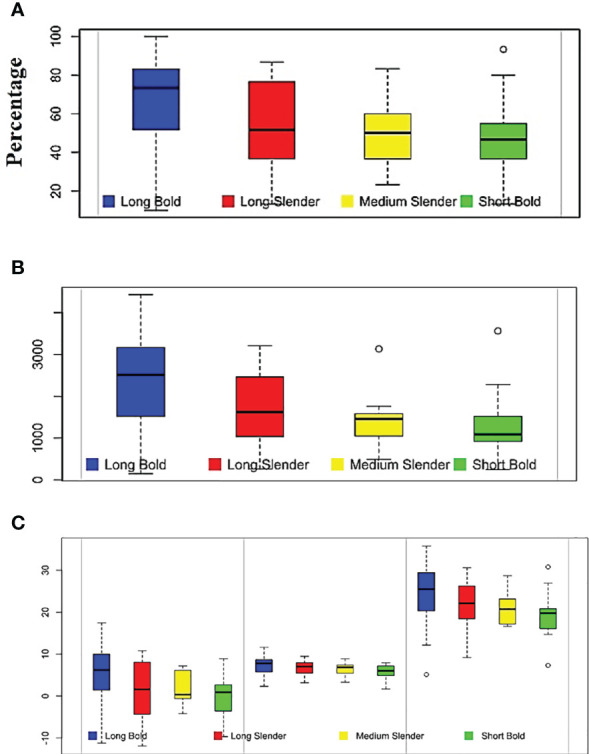
Boxplot showing the variability of anaerobic germination tolerance -associated traits among different grain types. **(A)** Anaerobic germination percentage (%), **(B)** anaerobic vigor index, and **(C)** from left to right, response index, root length (cm) and shoot length (cm).

In the present study, 119 genotypes were grouped on the basis of the grain’s physical traits and traits associated with AG potential. Trait means were compared among different grain-type groups and AGT groups (see **Table 5**). Among the grain-type groups, the study compared the means of the selected traits among long–bold, long–slender, medium–slender and short–bold groups. The landraces from the short–slender group were also included in the short–bold group, because both short–slender and short–bold groups had so few accessions (i.e.,< 10). The mean AGP of the long–bold landraces was 67.72%, which was significantly higher than the mean AGP of the other grain-type groups (47.72%). The long–bold grain type group also achieved a significantly higher AVI and shoot length than the rest of the grain type groups. Conversely, the lowest AGP and AVI values were recorded for the short–bold grain-type group (**Figure 2**). No significant difference was found among the landraces of different grain-type groups in terms of root length, shoot-to-root ratio, number of leaves, number of roots, fresh weight, dry weight, or response index. However, the tolerant and susceptible categories differed significantly in terms of AGP, AVI, SL, RL, RI, HSW, KB, and LBR. The accessions in the tolerant and moderately tolerant categories had an AGP of more than 78.98% and an AVI of 2816, which were significantly higher than the susceptible categories. Furthermore, the accessions in the susceptible group had an AGP of 30.80% and an AVI of 801. The shoot length, root length, response index, and grain weight of the landraces in the tolerant and moderately tolerant groups were significantly different from those of the susceptible groups. Likewise, the kernel breadth and LBR values of the tolerant group were significantly different from the susceptible categories.

**Table 5:** Mean comparison of grain type and tolerance reaction of 119 rice landraces by anaerobic germination tolerance-associated trait.

Trait	Grain type				Tolerance reaction			
	Long–bold	Long–slender	Medium–slender	Short–bold/slender	Tolerant	Moderately tolerant	Moderately susceptible	Susceptible
**AGP (%)**	67.72 ± 20.79 a	54.44 ± 21.95 b	48.97 ± 15.66 b	47.72 ± 19.50 b	93.64 ± 3.48 a	78.98 ± 4.35 b	54.65 ± 7.17 c	30.80 ± 9.07 d
**AVI**	2,331.08 ± 1,033.8 a	1,736.66 ± 876.24 b	1,441.85 ± 625.39 b	1,324.00 ± 733.68 b	3,672.86 ± 456.41 a	2,816.15 ± 359.79 b	1,567.74 ± 382.68 c	801.46 ± 292.12 d
**SL (cm)**	24.59 ± 6.44 a	21.85 ± 5.56 ab	20.89 ± 3.94 ab	19.24 ± 4.99 b	30.18 ± 3.10 a	27.33 ± 3.70 b	21.26 ± 4.14 c	16.57 ± 4.48 d
**RL (cm)**	7.36 ± 2.20 a	6.63 ± 1.84 a	6.39 ± 1.75 a	5.82 ± 1.73 a	8.88 ± 0.88 a	8.26 ± 1.44 a	6.63 ± 1.66 b	4.70 ± 1.43 c
**RI**	5.09 ± 6.89 a	0.89 ± 7.20 a	1.68 ± 4.08 a	–0.06 ± 5.60 a	8.46 ± 5.81 a	6.78 ± 4.91 a	1.93 ± 6.41 b	–1.98 ± 5.87 c
**SRR**	3.87 ± 1.29 a	4.03 ± 2.19 a	3.89 ± 1.14 a	3.52 ± 1.17 a	3.79 ± 0.70 a	3.95 ± 1.18 a	3.90 ± 1.88 a	3.67 ± 1.36 a
**NOL**	1.91 ± 0.25 a	1.91 ± 0.27 a	1.88 ± 0.31 a	1.76 ± 0.36 a	2.06 ± 0.24 a	1.97 ± 0.24 a	1.90 ± 0.19 a	1.69 ± 0.36 b
**NOR**	6.29 ± 1.82 a	6.01 ± 1.55 a	6.04 ± 1.71 a	6.28 ± 1.26 a	7.03 ± 1.92 a	6.15 ± 1.50 ab	6.38 ± 1.74 ab	5.70 ± 1.55 b
**FW (g)**	0.14 ± 0.05 a	0.14 ± 0.07 a	0.16 ± 0.09 a	0.16 ± 0.06 a	0.13 ± 0.04 a	0.14 ± 0.04 a	0.15 ± 0.06 a	0.16 ± 0.09 a
**DW (g)**	0.09 ± 0.05 a	0.08 ± 0.03 a	0.08 ± 0.04 a	0.10 ± 0.05 a	0.08 ± 0.03 a	0.08 ± 0.03 a	0.09 ± 0.05 a	0.09 ± 0.05 a
**KL (mm)**	6.98 ± 0.97 a	7.07 ± 0.93 a	5.63 ± 0.23 b	5.45 ± 0.58 b	6.45 ± 0.90 a	6.94 ± 1.03 a	6.51 ± 1.10 a	6.39 ± 1.11 a
**KB (mm)**	2.69 ± 0.38 a	2.07 ± 0.18 b	2.08 ± 0.14 b	2.11 ± 0.34 b	2.64 ± 0.38 a	2.52 ± 0.42 ab	2.37 ± 0.44 ab	2.22 ± 0.42 b
**LBR**	2.60 ± 0.21 b	3.42 ± 0.28 a	2.72 ± 0.15 b	2.65 ± 0.51 b	2.46 ± 0.22 b	2.79 ± 0.44 a	2.79 ± 0.44 a	2.91 ± 0.39 a
**HSW (g)**	2.63 ± 0.51 a	2.14 ± 0.54 b	1.82 ± 0.54 b	2.09 ± 0.83 b	2.58 ± 0.25 a	2.58 ± 0.57 a	2.32 ± 0.68 ab	2.06 ± 0.68 b

The means followed by the same letters are not significant at a *p*-value ≥ 0.05 and the means followed by different letters are significant at a *p*-value ≤ 0.05. AGP, anaerobic germination percentage; SL, shoot length; RL, root length; RI, response index; SRR, shoot-to-root ratio; NOL, number of leaves; NOR, number of roots; FW, fresh weight; DW, dry weight; AVI, anaerobic vigor index; KL, kernel length; KB, kernel breadth; LBR, length-to-breadth ratio; HSW, 100-seed weight.

### Correlation

**Figure 3:**
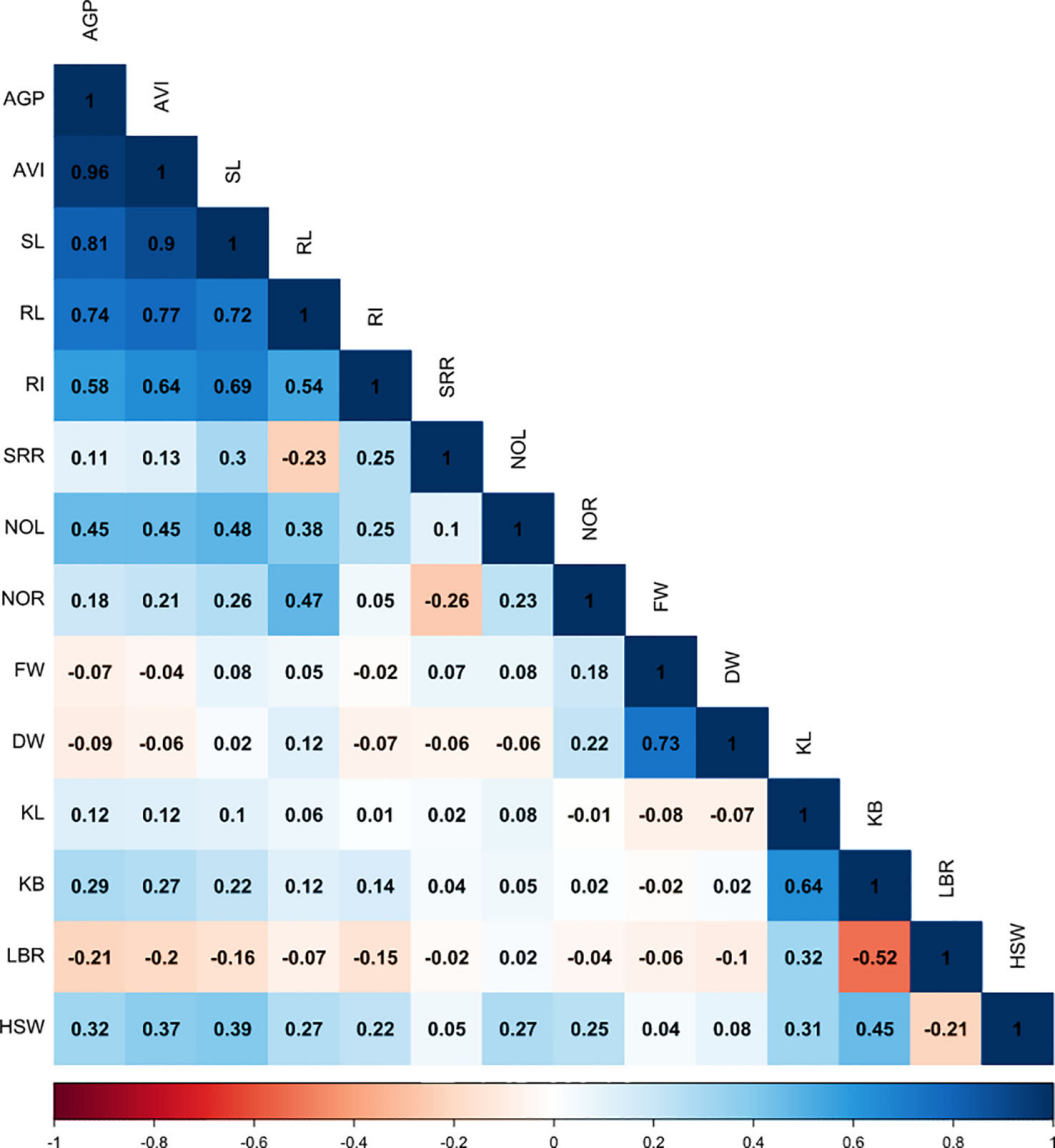
Correlogram showing association between grain morphology traits and traits associated with anaerobic germination potential. AGP, anaerobic germination percentage; SL, shoot length; RL, root length; SRR, shoot-to-root ratio; NOL, number of leaves; NOR, number of roots; FW, fresh weight; DW, dry weight; AVI, anaerobic vigor index; RI, response index; KL, kernel length; KB, kernel breadth; LBR, length-to-breadth ratio; HSW, hundred-seed weight.

In this study, a simple correlation analysis was conducted between the seedlings’ morphological traits and the early-stage submergence tolerance traits, such as AGP and AVI, among the genotypes under study. The AGP and AVI (*r* = 0.96***, *p*< 0.001) values were found to be significantly and positively associated with RI, shoot and root length, number of leaves and roots, grain breadth, and HSW. However, a positive and non-significant association was observed with grain length (**Figure 3**). Shoot length had a strong positive association with root length (*r* = 0.72***, *p*< 0.001) under hypoxic conditions. A significant negative correlation was observed between grain length–breadth ratio and AGP (*r* = –0.21*, *p*< 0.05) and AVI (*r* = –0.20*, *p*< 0.05). Furthermore, LBR exhibited a strong negative association with kernel breadth (*r* = –0.52***, *p<* 0.001) and grain weight, and a positive significant correlation with grain length. A highly significant association under anaerobic treatment was found between RI and the following traits: SRR, shoot length, and number of leaves. The association among shoot length, root length, SRR, and number of roots was significant and positive. Similarly, both grain length and breadth were positively correlated with grain weight.

### Principal component analysis

**Figure 4:**
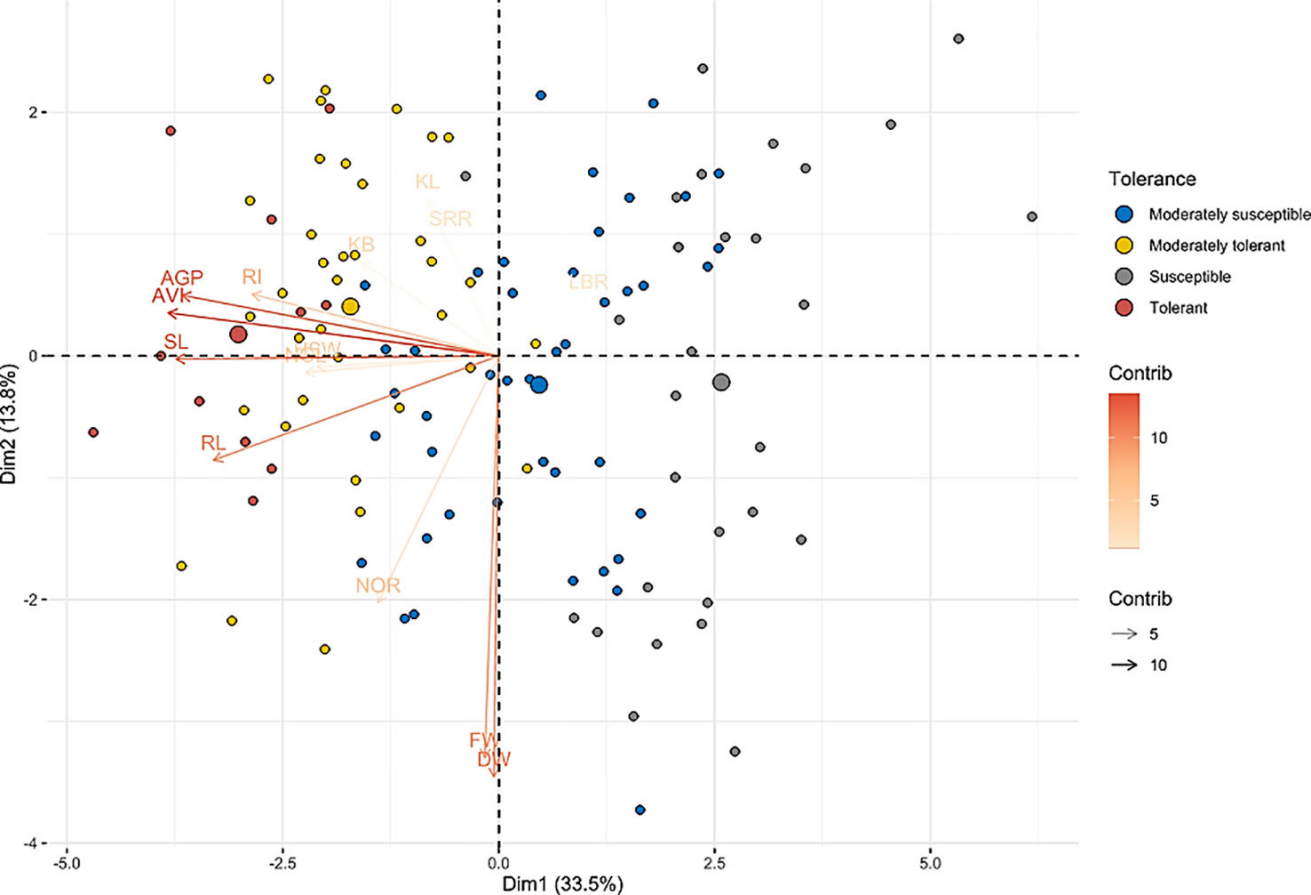
Biplot principal component analysis of native rice landraces for principal component 1 vs. principal component 2. The biplot depicts the distribution patterns of indigenous accessions into four different quadrants based on the traits associated with anaerobic germination tolerance and clearly separating tolerant genotypes from susceptible genotypes. Color gradients represent the contribution of variables toward the phenotypic variance of the corresponding principal component. **Supplementary Figure 2** shows the exact position of each genotype on the biplot.

The phenotypic data for 14 morphological traits under hypoxia was used to conduct the principal component analysis to assess the contribution of the individual traits to total variation and to analyze the genetic variations among the native landraces. The first five principal components (PCs) with an eigenvalue of > 1 accounted for 79.15% of the total variation (**Supplementary Figure 1**). Among them, PC1, with an eigenvalue of 4.69, accounted for 33.53% of the variation followed by PC2, which accounted for 13.82% of the variation (**Table 6**). In PC1, AVI exhibited the highest positive value with a loading of 0.892, followed by shoot length (0.850), AGP (0.819), root length (0.664), and RI (0.497). Traits associated with AG potential, such as AVI, AGP, RI, shoot and root length, and grain weight, were key traits contributing to the total variation exhibited by PC1. In PC2, both fresh and dry weight contributed to the high variation. KL, KB, and HSW for PC3 and SRR for PC4, accounted for 0.503% and 10.24% of the total variation, respectively. Variations contributed by PC5 (9.06%) were driven by LBR. The results of the loading and biplot analysis revealed that traits such as AGP, AVI, RI, shoot length, root length, HSW, and grain breadth were crucial traits that contributed to the total variability of the landraces, whereas the rest of the traits contributed minimally toward the phenotypic variability (**Figure 4**).

**Table 6:** Principal components of traits associated with anaerobic germination potential for rice landraces studied under anaerobic stress.

Parameters	PC1	PC2	PC3	PC4	PC5
**Eigenvalue**	**4.69**	**1.93**	**1.75**	**1.43**	**1.27**
**Variance (%)**	**33.53**	**13.82**	**12.50**	**10.24**	**9.06**
**Cumulative**	**33.53**	**47.35**	**59.86**	**70.09**	**79.15**
**AGP**	**0.819**	0.015	0.017	0.000	0.004
**AVI**	**0.892**	0.008	0.020	0.001	0.001
**SL**	**0.850**	0.000	0.022	0.020	0.016
**RL**	**0.664**	0.045	0.048	0.095	0.013
**RI**	**0.497**	0.016	0.045	0.073	0.001
**SRR**	0.019	0.059	0.001	**0.503**	0.225
**NOL**	0.304	0.001	0.025	0.016	0.067
**NOR**	0.119	0.250	0.001	**0.240**	0.018
**FW**	0.002	**0.663**	0.025	0.073	0.098
**DW**	0.000	**0.727**	0.053	0.027	0.029
**KL**	0.041	0.101	**0.417**	0.144	0.233
**KB**	0.154	0.036	**0.721**	0.003	0.025
**LBR**	0.066	0.013	0.112	0.230	**0.536**
**HSW**	0.269	0.001	**0.245**	0.008	0.001

PC, principal component; AGP, anaerobic germination percentage; AVI, anaerobic vigor index; SL, shoot length; RL, root length; RI, response index; SRR, shoot-to-root ratio; NOL, number of leaves; NOR, number of roots; FW, fresh weight; DW, dry weight; KL, kernel length; KB, kernel breadth; LBR, length-to-breadth ratio; HSW, 100-seed weight. Bold values indicates the major contribution of corresponding variables to the particular principal component.

Biplot analysis between PC1 and PC2 explains the distribution and nature of the diversity based on the variables as well as genotypes (**Figure 4** and **Supplementary Figure 2**). The landraces considered in this study were efficiently divided into four quarters by the biplot of first two PCA components. These in turn divided most of the AG-resistant and susceptible (IR42) landraces into distinct quadrants. Highly tolerant landraces, such as *Karuthakar, Edakkal*, and *Varappu kudainchan*, were clustered within the first quadrant (top left), whereas *Poovan samba, Mattaikar, Mandamaranellu*, and *Manvilayan* were grouped within the fourth quadrant (bottom left). Furthermore, cultivars ‘IR42’, ‘FR13 A’, ‘CO 43’, and ‘CO 43 Sub1’ were placed in the second quadrant (top right). The first and the fourth quadrants together comprised all of the tolerant and moderately tolerant landraces, except *Karnel* (second quadrant) and *Chittimutiyalu* (third quadrant), with high AGP, AVI, RI, and shoot and root length values along with an LBR ratio of > 2.5. The second and third quadrants encompassed the majority of the susceptible landraces, including ‘IR 42’. Landraces such as *Samba Massanam* (10%), *Maranellu* (13.33%), and *Salem samba* (13.33%) were highly susceptible and belonged to the most divergent cluster in the second quadrant, which distinguishes them from the rest of the landraces.

### Cluster analysis

**Figure 5:**
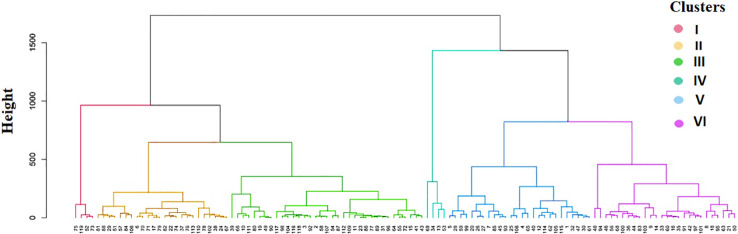
Dendrogram showing similarity index for the 119 native landraces based on traits associated with anaerobic germination potential. Colored branches indicate clusters I to VI (from left to right).

The Ward.D2 cluster analysis method was applied to the indigenous rice landraces based on the traits associated with AG potential to group the landraces into six distinct clusters (**Figure 5**). The number of landraces in each group varied between four in clusters I and IV to 35 in cluster III (**Table 7**). Cluster IV comprised four tolerant landraces *viz.*, *Karuthakar*, *Poovan samba, Mattaikar* and *Edakkal*, with the highest mean values for AGP (97.50%), AVI (4,200.28), SL (33.36 cm), RL (9.53 cm), RI (12.82), NOL (2.20), and NOR (6.95) compared with other clusters. Cluster VI, included 19 moderately tolerant landraces and seven tolerant landraces (≥ 90% AGP), such as *Manvilayan*, *Mandamaranellu*, *Varappu kudainchan, Varisuriyan, Mohini samba, Katta samba*, and *Kaan*, possessed a high mean value for SRR (4.29). The majority of landraces with high values for AGP-associated traits were in clusters IV and VI. These landraces possessed high values for AGP (> 73.33%), AVI (2,834–4,433), shoot length (25.11–35.76 cm), and RI. Cluster V had 26 landraces, 17 of which were identified as moderately tolerant (> 71% of AGP) and nine as moderately susceptible. Cluster III had 35 genotypes, forming the largest cluster, with an AGP of 16.67%–56.67%, and including ‘FR13 A’, ‘CO 43’, and ‘CO 43 Sub1’. Cluster II included 24 landraces, whereas susceptible control variety ‘IR 42’ clustered with other susceptible landraces *Maranellu, Salem samba*, and *Samba masanam* in cluster I. The mean values of each cluster significantly differed for all traits, except kernel length, kernel breadth, and grain weight. On average, the phenotypic within-cluster distance was found to be highest in cluster IV, followed by clusters I and II; cluster V had the lowest mean distance (**Supplementary Table 3**). The highest average distance was observed between clusters V and VI, followed by clusters II and VI; the lowest distance was observed between clusters II and III.

**Table 7:** Mean comparison of anaerobic germination tolerance-associated traits among the 119 indigenous rice landraces grouped in five clusters.

	Cluster I (*n* = 4)	Cluster II (*n* = 24)	Cluster III (*n* = 35)	Cluster IV (*n* = 4)	Cluster V (*n* = 26)	Cluster VI (*n* = 26)
**AGP (%)**	12.50 e	53.75 c	38.10 d	97.50 a	71.92 b	83.97 b
**AVI**	234.56 f	1,581.17 d	936.17 e	4,200.28 a	2,358.12 c	3,179.76 b
**SL (cm)**	8.48 d	22.04 b	17.29 c	33.36 a	24.62 b	29.57 a
**RL (cm)**	2.76 d	6.83 b	4.93 c	9.53 a	8.19 ab	8.38 ab
**RI**	–4.64 d	2.41 bc	–2.16 cd	12.82 a	5.65 b	7.72 ab
**SRR**	2.05 b	4.06 a	3.84 a	3.97 a	3.47 a	4.29 a
**NOL**	1.27 c	1.91 ab	1.79 b	2.20 a	1.91 ab	2.01 ab
**NOR**	3.68 b	6.56 a	5.88 a	6.95 a	6.47 a	6.31 a
**FW (g)**	0.07 b	0.15 ab	0.16 a	0.13 ab	0.14 ab	0.14 ab
**DW (g)**	0.04 b	0.09 ab	0.10 a	0.07 ab	0.09 ab	0.08 ab
**KL (mm)**	7.05 a	6.52 a	6.28 a	6.15 a	6.71 a	7.02 a
**KB (mm)**	2.18 a	2.28 a	2.33 a	2.55 a	2.44 a	2.60 a
**LBR**	3.24 a	2.90 ab	2.74 ab	2.45 b	2.79 a	2.72 a
**HSW (g)**	2.18 a	2.25 a	2.06 a	2.66 a	2.47 a	2.71 a

The means followed by the same letters are not significant at a *p*-value ≥ 0.05 and the means followed by different letters are significant at a *p*-value ≤ 0.05. Values in parenthesis represent the number of landraces present in each cluster. AGP, anaerobic germination percentage; SL, shoot length; RL, root length; RI, response index; SRR, shoot-to-root ratio; NOL, number of leaves; NOR, number of roots; FW, fresh weight; DW, dry weight; AVI, anaerobic vigor index; KL, kernel length; KB, kernel breadth; LBR, length-to-breadth ratio; HSW, hundred-seed weight.

### Molecular diversity and clustering

**Figure 6:**
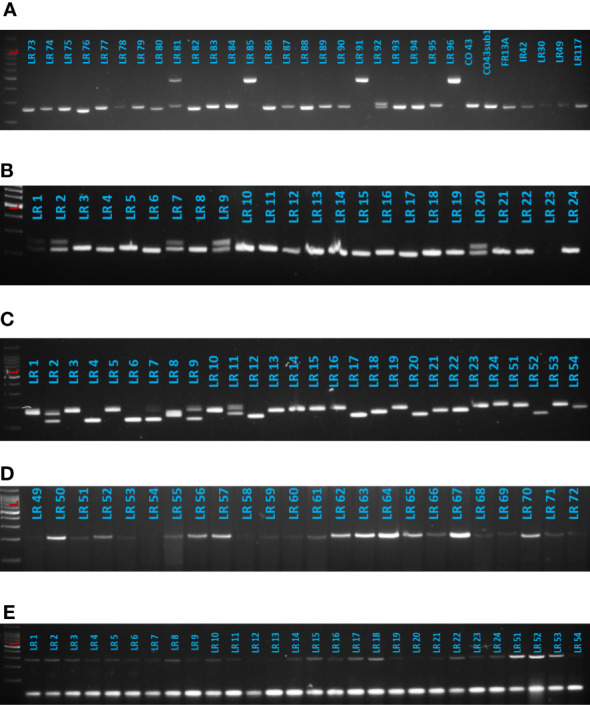
Representation of gel images of gene-linked markers. **(A)** DFR, **(B)** TTP_G4, **(C)** RM 206, **(D)** RM 478, and **(E)** RM 24161.

Thirteen alleles ranging from two to four alleles per locus were detected with the four gene-specific markers linked to AG tolerance (**Figure 6**). The average number of alleles per locus was 3.25. The PIC value for each marker locus enabled us to understand the polymorphism level among the landraces; PIC values ranged from 0.359 to 0.681, with an average of 0.496 per locus (**Table 8**). Marker RM 24161 was monomorphic among the studied landraces.

**Table 8:** Genetic diversity parameters of anaerobic germination tolerance-specific markers.

Marker	Gene/QTL	Chromosome number	Na	MAF	PIC
**RM 478**	*AG2*	7	2	0.61	0.359
**TTP_G4**	*AG1*	9	3	0.52	0.431
**DFR**	*AG1*	9	4	0.44	0.511
**RM 206**	*qAG11*	11	4	0.31	0.681
**Mean**			3.25	0.47	0.496
**Maximum**			4	0.61	0.681
**Minimum**			2	0.31	0.359

Na, number of alleles; MAF, major allele frequency; PIC, polymorphic information content.

We conducted a single-factor ANOVA-based single-marker analysis to validate genetic associations among four polymorphic markers and the traits associated with AG potential. Statistically, five significant genetic associations were observed for four traits (SRR, KB, LBR, and HSW) (**Table 9**). These five significant marker–trait associations (MTAs) accounted for 6.28%–19.46% of the total observed phenotypic variance (*R* ^2^). SRR showed a significant association (*p*< 0.05) with RM478 (*R* ^2^ = 6.28%). Similarly, KB and LBR showed a highly significant association (*p*< 0.01) with RM206. HSW had a significant genetic association with two polymorphic markers (RM478 and RM206).

**Table 9:** Summary statistics of marker trait association analysis.

Marker	*p*-value				*R* ^2^ (%)
	DFR	TTP_G4	RM478	RM206	
AGP	0.164	0.118	0.655	0.885	–
AVI	0.149	0.080	0.662	0.551	–
SL	0.659	0.394	0.670	0.595	–
RL	0.524	0.487	0.229	0.407	–
RI	0.205	0.403	0.475	0.102	–
SRR	0.672	0.833	**0.023**	0.880	6.28
NOL	0.406	0.576	0.577	0.462	–
NOR	0.551	0.800	0.406	0.166	–
FW	0.856	0.227	0.545	0.843	–
DW	0.911	0.294	0.660	0.824	–
KL	0.967	0.682	0.335	0.140	–
KB	0.450	0.410	0.611	**8.00 E^-05^**	19.46
LBR	0.787	0.293	0.742	**0.001**	14.28
HSW	0.247	0.499	**0.013**	**0.011**	7.22 (RM478) 9.93 (RM206)

Bold value indicates the significant p values.

The 119 genotypes considered for the investigation were grouped into four clusters ranging from 16 landraces (cluster II) to 49 landraces (cluster IV) (**Supplementary Figure 3**). Cluster I included 33 landraces, whereas cluster III included 21. Cluster II included the majority of susceptible landraces, including ‘IR 42’ and ‘FR 13A’. *Karuthakar* and *Poovan samba*, in cluster III, formed a distinct sub-cluster. Cluster IV displayed a higher mean distance (3.73). Inter-cluster distance was highest between clusters I and IV, followed by the clusters I and III (**Table 10**).

**Table 10:** Mean Nei distance within and among the five clusters of rice landraces, grouped by AGP.

Cluster	1	2	3	4
**1**	2.98			
**2**	3.02	3.00		
**3**	3.52	2.85	3.50	
**4**	3.54	3.39	3.44	3.73

## Discussion

### Variability components associated with anaerobic germination

Rice is widely consumed across the globe, accounting for 43% of overall food grain production in globe and 46% of cereal production in India (Mohanapriya et al., 2022). Rice productivity is heavily affected by abiotic stressors; thus, climate change and global warming present a risk to this important food source. Flooding is a serious problem that can cause complete crop failure. Within one decade, the flood-affected landmass in India increased from 19 to 40 Mha, i.e., from 12% to 25% of the total cultivable landmass (Kuanar et al., 2017). Deep-water, rain-fed lowland ecosystems constitute approximately 50 million hectares of land worldwide and account for one-third of global rice production areas (Oladosu et al., 2020). Thus, screening rice cultivars for AGT identifies early-stage flood-tolerant genotypes and their genetic potential. In this study, a diverse panel of rice landraces originating in southern Indian were collected and were conserved at TRRI, Aduthurai, Tamil Nadu, India. From the genotypes, 119 were studied for AGT under hypoxic conditions to identify promising cultivars for AGT improvement.

The statistical analyses of this work revealed a significant and wide range of variation among all the traits associated with anaerobic germination potential, except the fresh and dry weights of the seedlings. However, morphological traits of the grain, such as KL, KB, LBR, and HSW, which are governed by additive genes, showed high heritability (> 95%), suggesting that selection would be effective based on these traits. Furthermore, Vikram et al. (2016) and Barik et al. (2019) reported heritability > 60% for grain physical traits among indigenous rice landraces. AGT-associated traits were found to be highly heritable, except for SRR and NOL, which were grouped under low heritability. This analytic output suggests that these traits are complex in nature and are highly influenced by the environment. Earlier studies also found that traits associated with anaerobic germination potential had moderate-to-high heritability (Darko Asante et al., 2021). Of the 14 traits studied, 10 had an *h* ^2^ > 0.60, indicating that phenotypic selection is effective for these traits under anaerobic stress. Traits such as RI, AVI, AGP, SL, RL, and HSW had a high genetic advance as a percentage of mean (GAM) coupled with high heritability. High heritability plus high genetic advance would be the best genotype selection indicators (Afrin et al., 2017). Thus, these traits should be given highest priority in improving anaerobic germination potential during crop breeding programs.

It is crucial to select rice genotypes with high seedling vigor for germination under anaerobic conditions in a DSR system. Among the 119 genotypes studied, the landraces *Karuthakar, Poovan samba, Mattaikar, Edakkal, Manvilayan, Mandamaranellu, Varappu kudainchan, Varisurian, Katta samba, Kaan*, and *Mohini samba* were found to have > 90% AGP, and are regarded as AGT landraces. Angaji et al. (2010); Barik et al. (2019), and Darko Asante et al. (2021) also studied rice accessions for AG tolerance. They reported that genotypes with > 90% AGP are AGT genotypes. The current investigation revealed considerable variations in AVI values (150–4,433) among the genotypes, which were greater than previous studies that reported ranges of 81–1,720 (Barik et al., 2019) and 0–870 (Mohanapriya et al., 2022) for AVI values. High AGP coupled with maximum AVI are the best indicators for AGT in rice (Mohanapriya et al., 2022). Indigenous landraces such as *Karuthakar, Poovan samba, Mattaikar*, and *Edakkal* were found to possess high AGP and AVI values.

**Figure 7:**
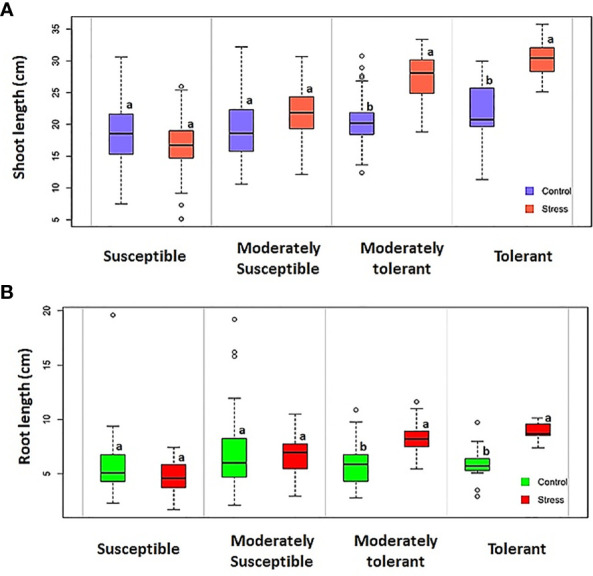
Boxplot depicting the shoot and root length of different tolerance group genotypes under controlled and hypoxia conditions. **(A)** Shoot length and **(B)** root length. The same letters on boxplots are not significant at *p* ≥ 0.05 while different letters are significant at *p* ≤ 0.05 based on the Newman–Keuls test.

This study observed significant differences between the control and experimental groups in terms of growth characteristics under hypoxic conditions. Susceptible landraces exhibited a significant reduction in both shoot and root length under hypoxia when compared with the tolerant landraces (**Figure 7**). Similar results were also obtained for the fresh and dry weights of the seedlings. Tolerant landraces possessed significantly higher shoot lengths under stress than the control genotypes. Shoot elongation is the result of a switch from high energy-demanding cell division to low energy-demanding cell growth (Atwell et al., 1982). During this phenomenon multiple processes occur, including the synthesis of cell walls and the uptake of solutes, which are less energy-demanding than protein synthesis. This might be due to the production of ethylene, which in turn triggers rapid shoot elongation in the germinating seeds in order to escape the hypoxia condition (Ismail et al., 2009; Darko Asante et al., 2021). The elongating coleoptiles behave as snorkels; they come into direct contact with air and facilitate gas exchange, enabling the embryos to develop (Mondal et al., 2020). It was also noticed in this study that the landraces with longer shoots had higher AGP values. This condition is primarily due to the rapid elongation of the shoots under submergence; rapid shoot elongation has been identified as an indicator of AGT (Ismail et al., 2009; Barik et al., 2019; Darko Asante et al., 2021). The high mean values for response index expressed by the tolerant and moderately tolerant groups further substantiate the importance of shoot elongation among AGT groups. Susceptible landraces exhibited reduced root length under hypoxic conditions, whereas tolerant and moderately tolerant (AGP > 70%) landraces possessed significantly higher root length under hypoxia than the controls. *Edakkal*, *Karuthakar*, and *Varappu kudainchan* minimized root length reduction and expressed higher shoot length. AGP and AVI when compared among the other tolerant genotypes which were expressing root elongation under hypoxia. Genotypes ‘FR13 A’ and ‘CO 43’ with the *Sub1* locus recorded lower shoot length and RI than the AGT landraces. This finding infers that the *Sub1* gene introgressed genotypes that were unable to cope with submergence during the germination process, despite being tolerant to vegetative-stage submergence. *Sub1* locus-conferring genotypes undergo a quiescent strategy (Fukao et al., 2006) that conserves carbohydrates (Ella et al., 2003; Sarkar and Bhattacharjee, 2011; Darko Asante et al., 2021). These genotypes resume growth by activating and using ethylene production rather than shoot elongation after submergence subsides. Although the *Sub1* locus is not crucial for anoxic rice growth, it provides an insight into why submergence-stimulated elongation occurs in environments with oxygen (Magneschi and Perata, 2009). Thus, it is evident that the mechanisms of tolerance to submergence at the germination and vegetative stages differ.

The *per se* performance of indigenous landraces according to grain-type group and the tolerance group demonstrated differences in traits associated with anaerobic germination potential. Among the grain-type groups, the landraces from the long–bold category exhibited superior AGP and AVI compared with the other grain types. Similarly, tolerant and moderately tolerant landraces largely belonged to the bold grain type and had higher KB, LBR, and grain weight than susceptible landraces. This raises the possibility that differences in kernel breadth, linked to grain type, can affect anaerobic germination potential. Reports in the literature indicate that accessions with round-medium grain shape and with low apparent amylose content express higher shoot length and survival rates (Biselli et al., 2014; Nghi et al., 2019).

The highest association was observed between AGP and AVI, followed by shoot length with AVI and shoot length with AGP. Under anaerobic conditions, longer shoot length is associated with higher survival rates. Barik et al. (2019) and Darko Asante et al. (2021) reported a direct relationship between shoot length and survival percentage. Furthermore, due to a strong and positive correlation between these attributes, the enhancement of one trait improves the other. Among seed morphological traits, KB was positively associated with AGP and AVI, whereas LBR was negatively associated with AGP. Thus, an anaerobic vigor index would be a reliable selection tool for improving the anaerobic germination potential of rice genotypes.

Principal component analysis measures the contribution of each component to total variance (Sinha and Mishra, 2013). This measure can be used to identify traits with a significant impact on phenotypic variability (Ray et al., 2013). The current study revealed that the most discriminatory traits were AVI, AGP, shoot length, RI, KB, and grain weight. Earlier studies also reported traits such as AGP, AVI, and shoot length (Miro et al., 2017; Barik et al., 2019; Sudeepthi et al., 2020; Darko Asante et al., 2021), and fresh and dry weight (Barik et al., 2019; Sudeepthi et al., 2020) to be discriminating traits. Future landrace selection should be based on these traits to attain greater genetic gain in AGT breeding programs.

In this study, landraces were clustered on the basis of traits associated with anaerobic germination potential. The 119 rice genotypes were clustered into six different clusters containing between four (clusters I and IV) and 44 (cluster V) landraces each. Of the 47 tolerant landraces (> 70% AGP) identified, 30 (63.8%) were grouped in clusters I and VI. The landraces in clusters I and VI all exhibit long–bold grain types, except *Uppu milagai*, which exhibits medium–slender grains. This implies the possibility of a direct relationship between grain type and AGT. The clustering of indigenous landraces revealed that susceptible and tolerant landraces alike formed different distinct clusters. Our study discovered no duplicate genotypes, suggesting that landraces possess a high degree of difference in anaerobic germination potential (Barik et al., 2019). Hybridization would be effective if genotypes are selected from diverse clusters. Tolerant landraces studied here are good candidates for breeding programs attempting to develop rice varieties with AGT. These findings can also be exploited to generate novel recombinants for anaerobic germination potential; these in turn can reveal underlying genetic mechanisms and map the novel QTLs associated with AGT traits.

The two InDel and three SSR markers used in this study were selected based on earlier reports that investigated AGT (Kretzschmar et al., 2015; Reddy et al., 2015; Kim et al., 2019). Clustering based on molecular information grouped the studied landraces into four clusters, with all clusters comprising susceptible to tolerant landraces. MTAs offer clues regarding the presence of trait-linked QTLs/genes in diverse genetic backgrounds. In this research, single-marker analysis indicated significant (*p*< 0.05) associations for HSW with RM478 and RM206. Conversely, only the single marker RM206 could be significantly associated with KB and LBR. Higher phenotypic variance values of significant markers indicate that they control a considerable amount of genetic variation in grain dimensions and could be reliable genetic markers for the further improvement of rice grain shape. For AGT-associated traits, only SRR had a significant association with RM478 when compared with other traits. In marker-assisted breeding programs, a strong MTA is preferred over a weak MTA to effectively exploit that particular marker for trait improvement. Hence, further analysis using a diverse set of polymorphic markers dispersed over the entire genome or next-generation sequencing would help to identify strong MTAs for AGT-associated traits that could be exploited in future breeding programs to develop an AGT varieties.

### Promising trait-specific landraces

In this study, promising trait-specific landraces were identified for traits associated with anaerobic germination potential and were compared with grain type. The genotypes that were identified as having anaerobic germination potential registered significantly higher values for the corresponding traits than the overall mean. Among the studied landraces, *Karuthakar, Poovan samba, Mattaikar, Manvilayan, Edakkal*, and *Varappu kudainchan* are promising candidates for traits associated with anaerobic germination potential. Salient agronomic features of these AGT landraces are reported in **Supplementary Table 4**. Early seedling vigor analysis outcomes suggest that *Karuthakar*, *Poovan samba*, and *Mattaikar* possess significant sheath and seedling elongation characteristics (Akshaya et al., 2020). These accessions can be used as parental lines that can be developed and released as AG-tolerant cultivars in breeding programs. Most tolerant accessions identified as having traits associated with anaerobic germination potential belonged to the long–bold grain-type group.

## Conclusion

The development and use of AG-tolerant rice varieties has drawn considerable interest recently; such varieties will promote food supply and rice production stability during climate change and global warming. The outcomes of this study provide the possibility of improving rice cultivars with AGT, as these germplasms provide wider genetic variations for traits associated with anaerobic germination potential. Modern breeding tools based on sequencing approaches and genomic selection will enable crop breeders to target desirable traits in chromosomes, and identify and elucidate the genetic mechanisms at play. Genome-wide association studies would be the ideal way to study such a diverse panel of landraces. They also provide the possibility of discovering superior AG-tolerant alleles or novel genes, which may offer tolerance to early submergence during germination. This trait can reduce crop loss occurring after persistent flooding in flood-prone areas during monsoons. The current study identified novel sources of AGT in landraces *Karuthakar, Poovan samba, Mattaikkar, Edakkal*, and *Manvilayan*, which can be used as donors in future breeding programs. This may in turn pave the way for understanding the genetic mechanisms that underlie anaerobic germination tolerance.

## Data availability statement

The original contributions presented in the study are included in the article/**Supplementary Material**. Further inquiries can be directed to the corresponding authors.

## Author contributions

All authors listed have made a substantial, direct, and intellectual contribution to the work, and approved it for publication.
