# Supplementary material for: Unraveling the genetic potential of native rice (Oryza sativa L.) landraces for tolerance to early-stage submergence
Source: Front Plant Sci. 2023 May 18;14:1083177. doi: 10.3389/fpls.2023.1083177 (PMC10232957; doi:10.3389/fpls.2023.1083177)
Supplement: Supplementary file 1 [file DataSheet_1.docx]

Supplementary Figure1: Scree plot depicting Eigen values of PC components


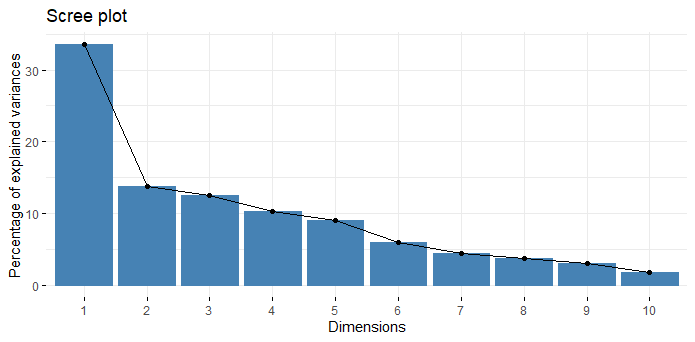


Supplementary Figure 2: Biplot analysis


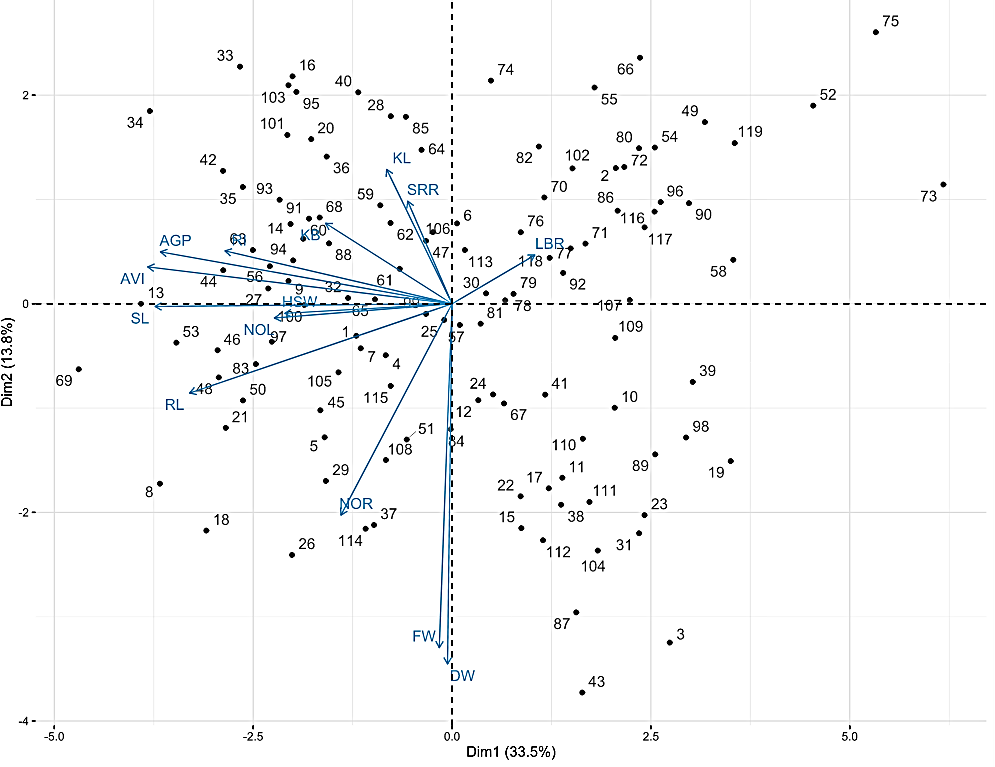


Supplementary Figure 3: Dendrogram using Nei distance matrix for native landraces based on molecular information


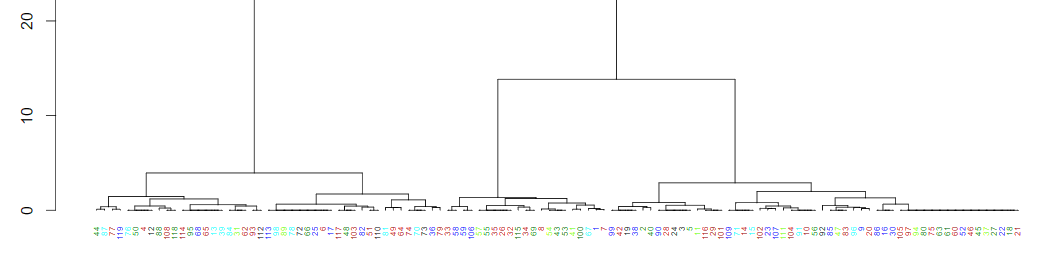


Supplementary Table 1: Order of the genotypes utilized in this study and pheno and geno cluster group of each landraces.

| Sl. No. | Genotypes | Pheno cluster group | Geno cluster group |
| --- | --- | --- | --- |
| 1 | Aanai komban | 5 | 4 |
| 2 | Aarupatham kuruvai | 3 | 3 |
| 3 | Aathur kichadi samba | 3 | 3 |
| 4 | Adukkan | 5 | 1 |
| 5 | Athur kichadi | 5 | 3 |
| 6 | Chandai kar | 2 | 1 |
| 7 | Chenellu | 5 | 4 |
| 8 | Chinkini kar | 6 | 4 |
| 9 | Chinna Punchai | 6 | 4 |
| 10 | Chinnar | 3 | 4 |
| 11 | Chithirai Kar | 3 | 3 |
| 12 | Chitti mutyalu | 5 | 1 |
| 13 | Edakkal | 4 | 1 |
| 14 | Gandakasala | 6 | 2 |
| 15 | GEB-24 | 3 | 2 |
| 16 | Gedumani | 6 | 4 |
| 17 | Illupai poo Samba | 2 | 1 |
| 18 | Iravai Pandi | 6 | 4 |
| 19 | Jai Sri Ram | 3 | 3 |
| 20 | Kuliadichan | 5 | 4 |
| 21 | Kaan | 6 | 4 |
| 22 | Kaatu Ponni | 2 | 4 |
| 23 | Kaatu samba | 3 | 4 |
| 24 | Kaivara samba | 2 | 3 |
| 25 | Kalanamak | 5 | 1 |
| 26 | Kaliyan Samba | 5 | 4 |
| 27 | Kallundai | 5 | 4 |
| 28 | Kandhasali | 5 | 3 |
| 29 | Karimbalan | 2 | 3 |
| 30 | Karnel | 5 | 4 |
| 31 | Karudan samba | 3 | 1 |
| 32 | Karuppu kavuni | 5 | 4 |
| 33 | Karuppu Nel | 6 | 3 |
| 34 | Karuthakar | 4 | 3 |
| 35 | Katta samba | 6 | 4 |
| 36 | Kattai kar | 6 | 1 |
| 37 | Kattu vanibam | 2 | 4 |
| 38 | Kichali samba | 2 | 3 |
| 39 | Kichadi samba | 3 | 1 |
| 40 | Koduvaliyan | 5 | 3 |
| 41 | Kothamalli samba | 3 | 4 |
| 42 | Kottanel | 6 | 3 |
| 43 | Kottara samba | 3 | 4 |
| 44 | Kudavaraghai | 6 | 2 |
| 45 | Kullakkar | 5 | 4 |
| 46 | Kuruvai kalanjiyam | 6 | 4 |
| 47 | Kuthala samba | 5 | 4 |
| 48 | Mandamaranellu | 6 | 1 |
| 49 | Manjal ponni | 3 | 2 |
| 50 | Manvilayan | 6 | 1 |
| 51 | Mappilai samba | 2 | 1 |
| 52 | Maranellu | 1 | 4 |
| 53 | Mattaikar | 4 | 4 |
| 54 | Melaki | 3 | 4 |
| 55 | Milagu samba | 3 | 4 |
| 56 | Mohini Samba | 6 | 4 |
| 57 | Mullampunchan | 2 | 4 |
| 58 | Mutrina Samba | 3 | 3 |
| 59 | Mysore malli | 5 | 3 |
| 60 | Navara | 6 | 4 |
| 61 | Nootripathu | 5 | 4 |
| 62 | Norungan | 5 | 1 |
| 63 | Ottadai | 6 | 4 |
| 64 | Ottadaiyan | 3 | 2 |
| 65 | Pal Kichadi | 5 | 1 |
| 66 | Pisini | 3 | 1 |
| 67 | Pommi | 2 | 4 |
| 68 | Poongar | 6 | 1 |
| 69 | Poovan samba | 4 | 3 |
| 70 | Rajamannar | 2 | 2 |
| 71 | Rasakatam | 2 | 2 |
| 72 | Rathasali | 3 | 1 |
| 73 | Salem samba | 1 | 2 |
| 74 | Salem sannam | 2 | 2 |
| 75 | Samba Massanam | 1 | 4 |
| 76 | Sanka samba | 2 | 1 |
| 77 | Savul samba | 3 | 2 |
| 78 | Seeraga samba | 2 | 1 |
| 79 | Sembalai | 2 | 2 |
| 80 | Sivappu chithiraikar | 3 | 4 |
| 81 | Sivappu malli | 2 | 1 |
| 82 | Sugandni samba | 2 | 1 |
| 83 | Thandi palliyan | 6 | 4 |
| 84 | Thanga samba | 2 | 1 |
| 85 | Thengai poo samba | 5 | 4 |
| 86 | Thillainayagam | 3 | 4 |
| 87 | Thirupathisaram | 3 | 2 |
| 88 | Thondi | 2 | 1 |
| 89 | Thulasi vasanai | 3 | 1 |
| 90 | Thuyamalli | 3 | 3 |
| 91 | Uppu Milagai | 6 | 4 |
| 92 | Vadan samba | 3 | 4 |
| 93 | Valan | 5 | 1 |
| 94 | Varappu kudainchan | 6 | 4 |
| 95 | Varisurian | 6 | 1 |
| 96 | Vasanai seeraga samba | 3 | 4 |
| 97 | Vasaramundan | 6 | 4 |
| 98 | Vellai chithirai kar | 3 | 1 |
| 99 | Vellai kavuni | 5 | 3 |
| 100 | Soora kuruvai | 6 | 4 |
| 101 | Sowattara samba | 6 | 3 |
| 102 | Chinna ponni | 2 | 4 |
| 103 | Koom vazhai | 6 | 1 |
| 104 | Altera | 3 | 4 |
| 105 | Madu muzhunki | 5 | 4 |
| 106 | Bhavani | 2 | 3 |
| 107 | Ganga | 3 | 4 |
| 108 | Jaya | 5 | 2 |
| 109 | Gopal bhog | 3 | 2 |
| 110 | Revathi | 2 | 1 |
| 111 | Saysree | 3 | 4 |
| 112 | Athira | 3 | 1 |
| 113 | Purple puttu | 2 | 1 |
| 114 | Mallikar | 5 | 1 |
| 115 | Sengalpattu sirumani | 5 | 4 |
| 116 | Co43 | 3 | 3 |
| 117 | Co43 sub1 | 3 | 1 |
| 118 | FR13A | 3 | 2 |
| 119 | IR42 | 1 | 2 |

Supplementary table 2: Grain type classification (Ramaiah, 1969)

| **Grain type** | **Length** | **LBR** |
| --- | --- | --- |
| Short Slender | <6.0 | >3.0 |
| Short Bold | <6.0 | <2.5 |
| Medium Slender | <6.0 | 2.5-3.0 |
| Long Slender | >6.0 | >3.0 |
| Long Bold | >6.0 | <3.0 |
| Extra Long Slender | >7.5 | >3.0 |
| Basmathi | >6.61 | >3.0 |

Supplementary table 3: Gower’s phenotypic distance (mean) within and among five clusters.

| **Cluster** | **1** | **2** | **3** | **4** | **5** | **6** |
| --- | --- | --- | --- | --- | --- | --- |
| **1** | 238.51 | 2244.15 | 1598.94 | 821.82 | 2946.19 | 1020.68 |
| **2** |  | 226.71 | 645.38 | 1422.46 | 702.22 | 3264.75 |
| **3** |  |  | 145.93 | 777.28 | 1347.38 | 2619.54 |
| **4** |  |  |  | 298.94 | 2124.51 | 1842.40 |
| **5** |  |  |  |  | 66.88 | 3966.76 |
| **6** |  |  |  |  |  | 208.65 |

Supplementary table 4. Agronomic features of AGT landraces

| **Landrace** | **DFF** | **DM** | **PH** | **NT** | **NPT** | **FL** | **FW** | **PL** | **SPY** |
| --- | --- | --- | --- | --- | --- | --- | --- | --- | --- |
| Karuthakar | 89 | 116 | 110.97 | 14.33 | 12.33 | 33.77 | 1.27 | 23.60 | 35.24 |
| Poovan samba | 117 | 131 | 153.07 | 15.00 | 13.33 | 41.40 | 1.37 | 28.93 | 32.14 |
| Mattaikar | 86 | 112 | 92.47 | 14.33 | 12.33 | 38.40 | 1.27 | 23.63 | 15.83 |
| Manvilayan | 92 | 125 | 115.67 | 15.33 | 14.33 | 29.60 | 1.10 | 22.73 | 31.17 |
| Edakkal | 83 | 115 | 124.13 | 23.67 | 20.33 | 37.57 | 1.07 | 31.17 | 26.17 |
| Varappu kudainchan | 93 | 125 | 104.50 | 20.00 | 17.00 | 34.90 | 1.23 | 22.33 | 24.55 |

DFF-Days to 50% flowering (days), DM-Days to maturity (days), PH-Plant height (cm), NT-Number of tillers, NPT-Number of productive tillers, FL-Flag leaf length, FW-Flag leaf width, PL-Panicle length, SPY-Single plant yield.
